# Improving Cytotoxicity of Saporin with Saponin SO1406 Isolated from the Roots of Saponaria Officinalis

**DOI:** 10.3390/biomedicines14030626

**Published:** 2026-03-11

**Authors:** Chaeeun Lim-Paik, Qinghua Zeng, Rebekah Beyea, Rebecca Boohaker, Pengfei Wang

**Affiliations:** 1 Department of Chemistry, University of Alabama at Birmingham, 901 14th Street South, Birmingham, AL 35294, USA; 2 Southern Research Institute, 2000 9th Avenue South, Birmingham, AL 35205, USA; 3 Adjuvax LLC, 2000 9th Avenue South, Birmingham, AL 35205, USA

**Keywords:** *Saponaria officinalis*, saponins, structure elucidation, saporin, cytotoxicity

## Abstract

**Background/Objectives**: Saponins have recently emerged as promising natural products that enhance toxin-based anticancer therapeutics by improving cytosol uptake. This study aimed to identify structurally defined novel natural saponins and evaluate their ability to enhance anticancer cytotoxicity. **Methods**: The roots of *Saponaria officinalis* L. were extracted with aqueous ethanol and purified by silica gel column chromatography and reverse-phase high-performance liquid chromatography (RP HPLC). The structures of new saponins were elucidated by NMR spectroscopy and mass spectrometry. Biological activity was assessed in vitro using multiple cancer cell lines. **Results**: Two pairs of structurally defined pure saponins were obtained: SO1406 and SO1448, and SO1684 and SO1726. Structural elucidation revealed that SO1684 and SO1726 share the core structure 3-*O*-*β*-D-Gal-(1→2)-[*β*-D-Xyl-(1→3)]-*β*-D-GlcA-gypsogenin-28-*O*-*β*-D-Qui-(1→4)-[*β*-D-Xyl-(1→3)-*β*-D-Xyl-(1→4)]-*α*-L-Rha-(1→2)-*β*-D-Fuc, with SO1684 acetylated at Qui O-4 and SO1726 bearing additional acetylation at Qui O-3. Deacetylation of either SO1684 or SO1726 afforded a known saponin SA1641 isolated from Saponinum album (Merck). Similarly, SO1406 and SO1448 were identified as 3-*O*-*β*-D-Gal-(1→2)-[*β*-D-Xyl-(1→3)]-*β*-D-GlcA-gypsogenin-28-*O*-*β*-D-Xyl-(1→4)-*α*-L-Rha-(1→2)-*β*-D-Fuc derivatives, each acetylated at Fuc O-4, with SO1448 containing an additional acetyl group at Fuc O-3. Among the isolated compounds, SO1684 is a known saponin and SO1406 exhibited the most pronounced biological activity, significantly enhancing the cytotoxicity of the ribosome-inactivating protein saporin (SAP) in the MDA-MB231 (triple-negative breast cancer) cell line. **Conclusions**: SO1406 demonstrates strong cytotoxicity-enhancing activity, highlighting the significant potential of structurally defined natural saponins to advance intracellular delivery and improve the therapeutic performance of protein-based anticancer agents.

## 1. Introduction

Saponins are amphiphilic glycosides widely distributed across various plant species and certain marine invertebrates. Many saponins exhibit intrinsic cytotoxic and immunomodulatory activities [1,2,3,4,5,6,7,8]. An underexplored aspect of saponins lies in their potential to augment the efficacy of anticancer drugs such as anticancer protein toxins [9,10,11,12,13,14,15,16,17,18,19,20,21]. Typically, protein toxins internalized into cells via receptor-mediated endocytosis become sequestered within endosomes. Only a small fraction of these internalized toxins can escape into the cytosol to exert their bioactivity, while the majority is ultimately degraded in lysosomes, substantially limiting their therapeutic potential.

To improve the cytosol uptake of protein toxins, several types of endosomal escape enhancers (EEEs) have been investigated, including those inspired by viral endosomal escape domains (EEDs) [22] and small molecules such as chloroquine, cyclosporin A, retinoic acid, and monensin [23,24,25,26,27]. Compared with these EEEs with only modest enhancement effects, natural saponins have recently emerged as a promising new class, demonstrating a remarkable ability to increase the pharmacological efficacy of toxin-based therapeutics [28].

**Figure 1:**
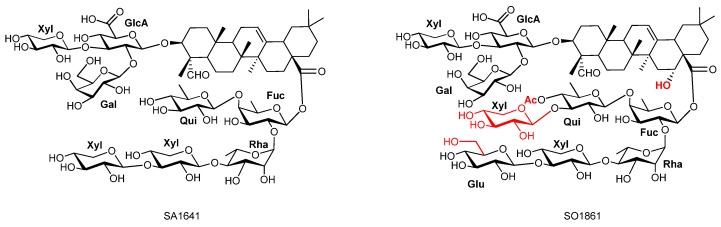
Structure of known SA1641 and SO1861.

While crude, undefined saponin mixtures have demonstrated their capacity in enhancing toxins’ cytotoxicity [12,13], they are unsuitable for clinical development, mechanistic studies, and structure–activity relationship (SAR) studies. It has been well established that the biological activity and synergistic potency of saponins are strongly dependent on their chemical structure [18,19,29,30]. However, only a limited number of structurally defined, pure natural saponins have been successfully isolated and systematically investigated to date [17,31,32,33,34,35,36]. Among them, SA1641 and SO1861 are two representative natural products (Figure 1). The former was isolated from Saponinum album (Merck, discontinued), a complex mixture of saponins extracted from *Gypsophila paniculata* L. [37], and the latter was isolated from the roots of *Saponaria officinalis* L. [20].

These two compounds markedly potentiate the cytotoxicity of toxins and their corresponding immunotoxins by facilitating their translocation from endosomal compartments into the cytosol [11,18,20,37,38,39,40,41,42,43,44]. In the present work, we describe our efforts to identify and characterize new structurally defined natural saponins capable of augmenting the cytotoxicity of anticancer toxins such as the ribosome-inactivating protein saporin (SAP).

SAP is a type I ribosome-inactivating protein (RIP-I) derived from the seeds of *Saponaria officinalis* [45,46]. It exerts cytotoxic N-glycosidase activity by depurinating specific adenine residues within the 28S rRNA of the 60S ribosomal subunit, thereby inhibiting protein synthesis. In contrast to type II ribosome-inactivating proteins (RIP-IIs), which comprise an enzymatically active A-chain and a B-chain responsible for cell binding and facilitating cytosolic uptake, A RIP-I consists solely of the A-chain. As a result, the internalization and subsequent endosomal escape of RIP-I such as SAP into the cytosol, where ribosomes reside, are inherently inefficient processes. To overcome this limitation and improve SAP’s therapeutic efficacy, various strategies have been investigated, including the use of EEEs [10,47].

## 2. Materials and Methods

### 2.1. General Experimental Procedures

Rotary evaporation at. ca. 12 Torr was used to concentrate the organic solutions. Thin-layer chromatography (TLC) was performed using glass plates pre-coated to a depth of 0.25 mm with 230–400 mesh silica gel impregnated with a fluorescent indicator (254 nm). The spots were visualized by ceric ammonium molybdate (CAM) stain. Column chromatography was performed using silica gel 60 (Silicycle). Reverse-phase high-performance liquid chromatography (RP HPLC) was run on a 1260 Infinity II (Agilent Technologies, Santa Clara, CA, USA) with a Prep C18, 250 × 10 mm, 5-micron column. NMR spectra (in CD_3_OD or C_5_D_5_N), including ^1^H, ^13^C, HMBC, HSQC, COSY, and TOCSY (t = 60 ms), were recorded on Bruker Avance III HD-600 and Avance III HD-850 spectrometers (Bruker, Billerica, MA, USA) equipped with TCI Cryoprobes. Chemical shifts (δ) are reported in ppm and were referenced to residual solvent resonances: for methanol-d_4_, δ_H_ 3.31 and δ_C_ 49.0 ppm; for pyridine-d5, δ_H_ 8.74, 7.58, and 7.22 and δ_C_ 150.35, 135.91, and 123.87 ppm. Spectra were processed in TopSpin 4.5. Deacetylation of the natural saponins was carried out in a methanol/water (1:1) solution at room temperature, and the pH was adjusted to 10–11 using 1.0 N NaOH.

### 2.2. Plant Samples

The roots of *Saponaria officinalis* L. (soapwort) were collected in Morocco and purchased from Health Embassy Ltd. (Cheltenham, Gloucestershire, UK). A voucher specimen (SO-EL-1-153) has been deposited at the Department of Chemistry, the University of Alabama at Birmingham, USA. Saporin was purchased from Advanced Targeting Systems (Carlsbad, CA, USA).

### 2.3. Extraction and Isolation

Soapwort roots (50 g) were ground and extracted with ethanol/water (500 mL, 1:1, *v*/*v*) at 30 °C overnight. The extract was filtered, and the filtrate was concentrated to give crude extract (20 g). The crude extract (15 g) was fractionated on a silica gel column, and eluted with chloroform/methanol/water (15:11:3) to provide semi-purified fraction A, 1.28 g and B, 6.27 g, after removal of the solvent. Fraction A (200 mg) was purified using C18 RP HPLC with acetonitrile/water gradients (0–3 min, 65:35, flow rate 10 mL/min; 3–20 min, 65:35, flow rate 25 mL/min; 20–20.05 min, 60:40, flow rate 25 mL/min; 20.05–26 min, 60:40, flow rate 25 mL/min) to provide SO1448 (*t*_R_ 15.5 min, 24.1 mg) and SO1726 (*t*_R_ 21.8 min, 34.6 mg). Another saponin SO1580, in between SO1448 and SO1726, was also isolated in a smaller amount and was not fully characterized. Fraction B (1.01 g) was re-fractionated with a silica gel column, and eluted with chloroform/methanol (3:2) and then chloroform/methanol/water (15:10:2) to afford a mixture (423 mg) with *R*_f_ 0.23–0.35 (chloroform/methanol/water 15:10:2). Part of this mixture (164 mg) was fractionated using C18 RP HPLC with acetonitrile/water gradients (0–8 min, 65:35, flow rate 25 mL/min; 8–13 min, 65:35, flow rate 15 mL/min; 13–14 min, 63:37, flow rate 15 mL/min) to provide fractions B1 (*t*_R_ 8.4 min, 103 mg) and B2 (*t*_R_ 10.9 min, 21 mg). B1 (103 mg) was re-fractionated using the same C18 RP HPLC with acetonitrile/water gradients (0–8 min, 65:35, flow rate 25 mL/min; 8–13 min, 65:35, flow rate 15 mL/min; 13–14 min, 63:37, flow rate 15 mL/min) to yield SO1406 (*t*_R_ 6.2 min, 25.7 mg), impure SO1538 (*t*_R_ 8.5 min, 28.2 mg), and SO1684 (*t*_R_ 10.5 min). Impure SO1684 (21.1 mg) was re-purified with a silica gel column, and eluted with chloroform/methanol CH_3_Cl/MeOH (3:2) and then chloroform/methanol/water (15:10:2) to obtain pure SO1684 (14.3 mg) as a white solid after removal of solvents. ESI–TOF MS of the extract also identified some other saponins with molecular weights of 1780, 1729, 1718, 1714, 1700, 1698, 1624, 1568, 1552, 1496, 1492, and 1364. Among them, SO1624, SO1580, SO1538, and SO1492 were isolated in an impure form, and their structures were not fully characterized with NMR studies.

### 2.4. Hemolysis Assay

Fresh donor rabbit RBC suspension (LAMPIRE Biological Laboratories, Fisher Scientific, Everett, PA, USA) was washed three times with PBS and diluted to 5%. Saponin samples were diluted to 1 mg/mL and then serially diluted in water to a final concentration of 7.8125 µg/mL. The samples and negative controls (water) were mixed with the 5% RBC suspension in 96-well plates at a 1:10 ratio by adding 20 µL of sample to 180 µL of RBC suspension. All samples and dilutions were prepared in duplicate for reproducibility. Plates were incubated for 30 min at 37 °C with high humidity and 5% CO_2_. After centrifugation, the supernatant was collected, and the hemoglobin content was measured at 562 nm. Percent hemolysis was calculated using VSA-2 (0.1 mg/mL) as the positive control and H_2_O (1:10) as the negative control. 

### 2.5. Cell Culture

The THP-1 cell line (ATCC TIB-202, Manassas, VA, USA) is a human monocytic cell line derived from a 1-year-old male with acute monocytic leukemia (AML). The RPMI 8226 cells (ATCC CRM-CCL-155, Manassas, VA, USA) are a human myeloma cell line derived from the peripheral blood of a 61-year-old male patient with multiple myeloma. The MDA-MB-231 cell line (ATCC CRM-HTB-26, Manassas, VA, USA) is a highly aggressive and invasive human breast cancer model derived from a pleural effusion of a 51-year-old Caucasian woman with triple-negative breast cancer (TNBC). The HepG2 cell line (ATCC HB-8065, Manassas, VA, USA) is a human liver cell line derived from a 15-year-old Caucasian male with a well-differentiated hepatocellular carcinoma. The FaDu cell line (ATCC HTB-43, Manassas, VA, USA) is a human hypopharyngeal squamous cell carcinoma line derived from a 56-year-old Caucasian male. THP-1 and RPMI 8226 were maintained in Roswell Park Memorial Institute 1640 Medium (RPMI-1640; Gibco, Thermo Fisher, 11875085, Grand Island, NY, USA) supplemented with 10% fetal bovine serum (FBS; PEAK Serum, PS-FB2, 24FD060, Bradenton, FL, USA). 2-Mercaptoethanol (Gibco, Thermo Fisher, 2198-023, Grand Island, NY, USA) was added freshly to a final concentration of 0.05 mM for THP-1. MDA-MB231, Hep-G2, and FaDu cells were maintained in Dulbecco’s Modified Eagle’s Medium (DMEM; Gibco 11965084, Grand Island, NY, USA) supplemented with 10% FBS (PEAK Serum, PS-FB2, 24FD060, Bradenton, FL, USA), at 37 °C in a 90% humidified atmosphere containing 5% CO_2_.

### 2.6. Cytotoxicity Assays

Cell viability was determined based on quantification of ATP using a Cell Titer-Glo Luminescent Cell Viability Assay (Promega G7572), which indicates the presence of metabolically active cells. Cells were seeded in 96-well plates at a density of 1 × 10^4^ cells/well in 50 mL of media and incubated overnight at 37 °C in a humidified atmosphere containing 5% CO_2_. On the following day, cells were treated with 50 mL of saporin, saponin, or their combinations at serial concentrations for 72 h. The plate and its contents were equilibrated at room temperature for approximately 30 min before adding 100 mL of Cell Titer-Glo reagent to the cells. The plate was incubated at room temperature for 10 min to stabilize the luminescent signal. Luminescence was acquired using a PHERAstar plate reader (BMG Labtech, Ortenberg, Germany). The 50% cytotoxic concentration (CC_50_) was determined by a nonlinear regression dose–response curve using GraphPad Prism (Version 10.1.2).

## 3. Results

### 3.1. Structure Elucidation

The roots of *Saponaria officinalis* L. were extracted with aqueous ethanol (1:1, *v*/*v*), followed by purification using silica gel chromatography and reverse-phase high-performance liquid chromatography (RP HPLC). This process yielded two pairs of structurally defined pure saponins: SO1684 and SO1726, and SO1406 and SO1448.

**Figure 2:**
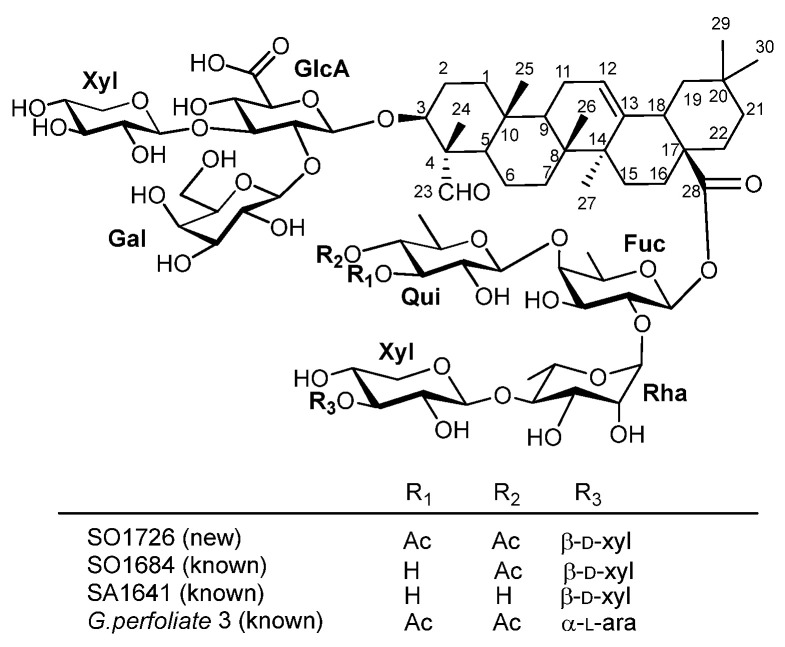
Structure of SO1726, SO1684, and known SA1641.

SO1726 was obtained as a white amorphous solid. HR-ESI–TOF MS showed its [M-H]^−^ at *m*/*z* 1725.7393 (*calc.* for C_79_H_121_O_41_, 1725.7383). The structure was elucidated by NMR collected in d_5_-pyridine (1D ^1^H and ^13^C; 2D TOCSY, COSY, HSQC, and HMBC; 3D NOESY-HSQC). The ^13^C NMR spectrum displayed 79 resonances corresponding to the aglycone (30 C), 8 sugar residues (47 C), and 2 acetyl carbonyls (2 C). In the ^1^H NMR spectrum, six tertiary methyl singlets were observed at δ_H_ 0.78, 0.83, 0.87, 1.02, 1.19, and 1.45 ppm, together with an olefinic proton resonance at δ_H_ 5.37 ppm (s, br). Consistent with ^13^C signals for six sp^3^-hybrid methyl carbons at δ_C_ 11.68 ppm (C-24), 16.18 ppm (C-25), 18.16 ppm (C-26), 26.27 ppm (C-27), 33.55 ppm (C-29), and 24.12 ppm (C-30), and for two sp^2^-hybrid carbons at δ_C_ 122.89 ppm (C-12) and 144.41 ppm (C-13), this confirmed an olean-12-ene skeleton (Figure 2). HSQC and HMBC established an oxymethine at C-3 (δ_H_ 4.05 ppm/δ_C_ 85.34 ppm), a methine at C-18 (δ_H_ 3.08 ppm, dd, *J* = 13.5, 3.6 Hz/δ_C_ 42.51 ppm), and a methylene at C-16 (δ_H_ 2.06 and 1.98 ppm/δ_C_ 23.62 ppm). A formyl singlet at δ_H_ 10.03 ppm (s) correlated with δ_C_ 211.58 ppm and showed HMBC to C-4 (δ_C_ 55.52 ppm) and C-24 (δ_C_ 11.68 ppm), placing the aldehyde at C-23. HMBC from H-18 (δ_H_ 3.08 ppm) to the ester carbon at δ_C_ 176.91 ppm located the carboxyl group at C-28. These findings suggested that the aglycone is gypsogenin (Figure 2).

Two-dimensional NMR (COSY, TOCSY, HSQC, HMBC, and NOESY-HSQC) identified the sugars as *β*-D-glucuronopyranosyl (GlcA), *β*-D-galactopyranosyl (Gal), three *β*-D-xylopyranosyl (Xyl), *β*-D-fucopyranoside (Fuc), *β*-D-quinovopyranosyl (Qui), and α-L-rhamopyranosyl (Rha). The D/L-configurations of the monosaccharides were assigned based on the fact that these sugar units with the particular configurations are the most encountered among the plant glycosides, especially in plants of the *Caryophyllaceae* family. The assignment was unambiguously confirmed by comparing ^1^H and ^13^C NMR data of deacetylated SO1726 with the published data of a known saponin, SA1641 (Tables S1 and S2) [16,17]. As shown in Figure 2, SO1726, SO1684, and SA1641 have the identical saponin skeleton; they differ only on the extent of acetylation. Deacetylated SO1726 and SO1684 have the same structure as SA1641; the D/L-configurations of the monosaccharides in SA1641 were experimentally determined. SA1641 was obtained from saponinum album (SA) of Merck, a complex mixture containing various saponins from roots of *Gypsophila* species of the Caryophyllaceae family. Interestingly, SA1641 was not observed in the crude mixture of the soapwort root extract based on MS analyses.

Eight anomeric proton signals were observed at δ_H_ 6.32 ppm (s, br, Rha), 5.97 ppm (d, *J* = 8.2 Hz, Fuc), 5.59 ppm (d, *J* = 7.5 Hz, Gal), 5.36 ppm (d, *J* = 9.0 Hz, Xyl(I)), 5.19 ppm (d, *J* = 7.5 Hz, Xyl (III)), 5.07 ppm (d, *J* = 7.8 Hz, Qui), 5.02 ppm (d, *J* = 7.4 Hz, Xyl (II)), and 4.87 ppm (d, *J* = 7.1 Hz, GlcA), correlating in HSQC to the eight peaks at δ_C_ 101.84, 94.79, 104.46, 105.50, 106.17, 106.23, 107.46, and 104.53 ppm, respectively, consistent with eight sugar residues (Table 1). The *β*-anomeric configuration for the D-GlcA, D-Gal, D-Xyl, D-Fuc, and D-Qui was indicated by ^3^*J*_H1-H2_ = 7–9 Hz; the *α*-anomeric configuration of L-Rha is consistent with its small anomeric coupling (^3^*J*_H1-H2_ = 1–3 Hz) and characteristic chemical-shift pattern.

At C-3 of gypsogenin aglycone, glycosylation was defined by HMBC from GlcA H-1 (δ_H_ 4.87 ppm) to C-3 (δ_c_ 85.34 ppm), with downstream linkages assigned by Gal H-1 (δ_H_ 5.59 ppm) to GlcA C-2 (δ_c_ 78.66 ppm) and Xyl (I) H-1 (δ_H_ 5.36 ppm) to GlcA C-3 (δ_c_ 86.58 ppm), giving a 3-*O*-[*β*-D-Gal-(1→2)][*β*-D-Xyl-(1→3)]-*β*-D-GlcA branched trisaccharide. A NOESY-HSQC cross-correlation between GlcA H-1 and C-3 further supported the 3-O linkage. The fragment-ion peak at *m*/*z* 939.5 observed with ESI–TOF MS also confirms the identity of gypsogenin connected with the trisaccharide moiety.

At C-28, HMBC from Fuc H-1 (δ_H_ 5.97 ppm) to the carboxyl carbon (δ_c_ 176.91 ppm) defined the point of attachment. The remaining sequence was established by Rha H-1 (δ_H_ 6.32 ppm, s) to Fuc C-2 (δ_c_ 74.62 ppm), Qui H-1 (δ_H_ 5.07 ppm) to Fuc C-4 (δ_c_ 84.28 ppm), Xyl (II) H-1 (δ_H_ 5.02 ppm) to Rha C-4 (δ_c_ 85.78 ppm), and Xyl (III) H-1 (δ_H_ 5.19 ppm) to Xyl (II) C-3 (δ_c_ 87.57 ppm), yielding a 28-*O*-*β*-D-Qui-(1→4)[*β*-D-Xyl-(1→3)-*β*-D-Xyl-(1→4)]-*α*-L-Rha-(1→2)-*β*-D-Fuc pentasaccharide. Two distinct acetyl methyl singlets at δ_H_ 2.06 and 1.98 ppm, together with HMBC from Qui H-3 (δ_H_ 5.64 ppm, t, *J* = 9.5 Hz) to a carbonyl at δ_c_ 170.83 ppm and from Qui H-4 (δ_H_ 5.08 ppm, t, *J* = 9.6 Hz) to a carbonyl at δ_c_ 170.56 ppm, established O-acetylation at Qui O-3 and O-4.

SO1684 was isolated from the same root extract as a white amorphous solid. HR-ESI–TOF MS showed its [M-H]^−^ at *m*/*z* 1683.7368 (*calc.* for C_77_H_119_O_40_, 1683.7278). SO1684 possesses the same structural backbone as SO1726, differing only by the presence of a single acetyl group at the Qui *O*-4 (Figure S1).

**Figure 3:**
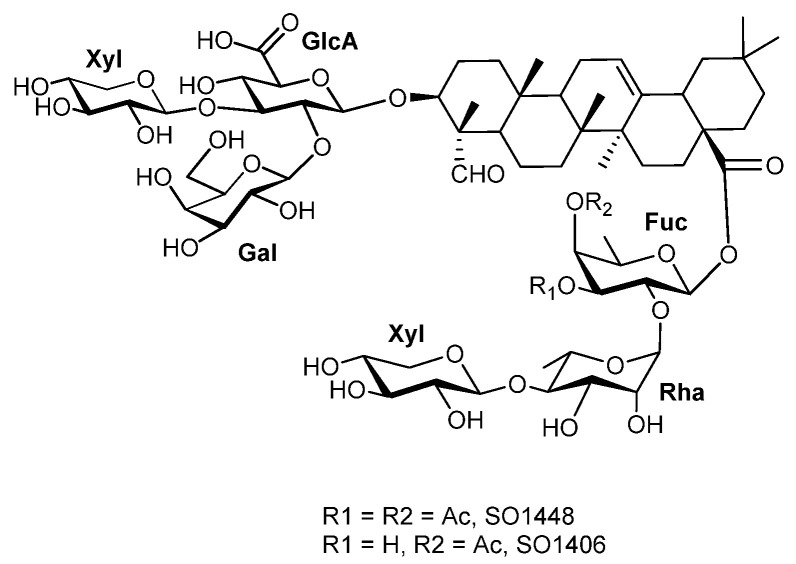
Structure of new saponins SO1406 and SO1448.

SO1406 was obtained as a white amorphous solid. Its molecular formula was determined by high-resolution electrospray ionization time-of-flight mass spectrometry in negative ion mode (HR-ESI–TOF MS), showing [M-H]^−^ *m*/*z* 1406.6273 (*calc.* for C_66_H_101_O_32_, 1405.6276). Its structure was elucidated with NMR studies, and all NMR data (1D ^1^H and ^13^C; 2D TOCSY, COSY, HSQC, and HMBC) were collected in d_4_-methanol. The ^13^C NMR spectrum displayed 67 resonances corresponding to the aglycone (30 C), 6 sugar residues (36 C), and 1 acetyl carbonyl (1 C) (Table 1). The ^1^H NMR spectrum showed six tertiary methyl singlets at δ_H_ 1.18 ppm (C-24), 0.99 ppm (C-25), 0.78 ppm (C-26), 1.17 ppm (C-27), 0.92 ppm (C-29), and 0.93 ppm (C-30), along with an olefinic proton resonance at δ_H_ 5.27 ppm (s, br, C-12) (Figure 1). These data were consistent with ^13^C signals for six sp^3^-hybridized methyl carbons at δ_C_ 10.96 ppm (C-24), 16.28 ppm (C-25), 17.82 ppm (C-26), 26.22 ppm (C-27), 33.49 ppm (C-29), and 24.12 ppm (C-30), and for the olefinic carbons at δ_C_ 123.40 ppm (C-12). The other olefinic carbon (C-13) is at 144.83 ppm. HSQC and HMBC established an oxymethine at C3 (δ_H_ 3.86 ppm/δ_C_ 86.44 ppm), a methine at C-18 (δ_H_ 2.82 ppm, dd, *J* = 13.5, 3.9 Hz/δ_C_ 42.86 ppm), and a methylene at C-16 (δ_H_ 2.05 and 1.68 ppm/δ_C_ 23.89 ppm). A formyl singlet at δ_H_ 9.45 ppm (s) correlated to δ_C_ 211.03 ppm and showed HMBC to C-4 (δ_C_ 56.23 ppm) and C-24 (δ_C_ 10.96 ppm), confirming the aldehyde at C-23. HMBC from H-18 (δ_H_ 2.82 ppm) to δ_C_ 178.02 located a carboxyl at C-28. These findings suggested the aglycone to be gypsogenin as well (Figure 3).

Six anomeric proton signals were observed at δ_H_ 5.40 ppm (d, *J* = 8.1 Hz, Fuc), 5.34 ppm (d, *J* = 1.4 Hz, Rha), 4.80 ppm (d, *J* = 7.0 Hz, Gal), 4.57 ppm (d, *J* = 7.7 Hz, Xyl (I)), 4.46 ppm (d, *J* = 7.4 Hz, GlcA), and 4.41 ppm (d, *J* = 7.7 Hz, Xyl (II)), correlating in HSQC with δ_C_ 94.99, 101.64, 103.76, 104.93, 104.58, and 107.48 ppm, respectively, indicating six sugar units. Two-dimensional NMR analyses further identified the sugars as *β*-D-GlcA, *β*-D-Gal, two *β*-D-Xyl, *β*-D-Fuc, and α-L-Rha. The β-anomeric configuration for the D-GlcA, D-Gal, D-Xyl, and D-Fuc was indicated by ^3^*J*_H1-H2_ = 7–9 Hz, while the α-anomeric configuration of L-Rha was supported by its small anomeric coupling constant and characteristic chemical-shift pattern.

At C-3 of gypsogenin aglycone, glycosylation was defined by HMBC from GlcA H-1 (δ_H_ 4.46 ppm) to C-3 (δ_c_ 86.44 ppm). Downstream linkages were assigned by correlations from Gal H-1 (δ_H_ 4.80 ppm) to GlcA C-2 (δ_c_ 78.11 ppm) and Xyl (I) H-1 (δ_H_ 4.57 ppm) to GlcA C-3 (δ_c_ 86.61 ppm), giving a 3-*O*-[*β*-D-galactopyranosyl-(1→2)][*β*-D-xylopyranosyl-(1→3)]-*β*-D-glucuronopyranosyl branched trisaccharide substitution.

At C-28 of gypsogenin aglycone, glycosylation was defined by HMBC from Fuc H-1 (δ_H_ 5.40 ppm) to the carboxyl carbon (δ_c_ 178.02 ppm). The remaining sequence was established by Rha H-1 (δ_H_ 5.34 ppm) to Fuc C-2 (δ_c_ 75.07 ppm), and Xyl (II) H-1 (δ_H_ 4.41 ppm) to Rha C-4 (δ_c_ 85.01 ppm), yielding a 28-*O*-*β*-D-xylopyranosyl-(1→4)-*α*-L-rhamopyranosyl-(1→2)-*β*-D-fucopyranosyl linear trisaccharide chain. A distinct acetyl singlet at δ_H_ 2.16 ppm, together with an HMBC correlation from Fuc H-4 (δ_H_ 5.08 ppm, d, *J* = 3.5 Hz) to a carbonyl at δ_c_ 172.78 ppm, established O-acetylation at Fuc O-4.

Another new saponin, SO1448, was also isolated from the same root extract. Its molecular formula was determined by mass spectrometry, with [M-H]^−^ at *m*/*z* 1447.6445 (*calc.* for C_68_H_103_O_33_, 1447.6382). It has the same structural skeleton as SO1406, except that it has an additional acetyl group attached to Fuc O-3, leading to a downfield shift of H-3 (δ_H_ 5.09 ppm, dd, *J* = 9.8, 3.5 Hz) (Figure S2). Deacetylation of SO1448 and SO1406 under mild alkaline conditions led to the identical deacetylated product based on ^1^H NMR analysis.

### 3.2. Hemolytic Activity

**Figure 4:**
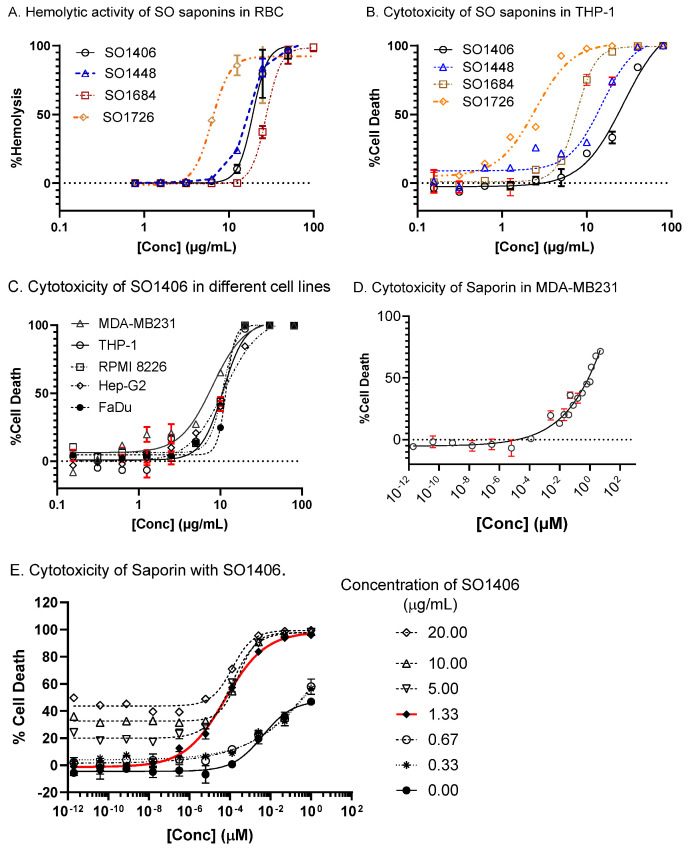
(**A**) Hemolytic activity of SO saponins in rabbit RBCs. (**B**) Cytotoxicity of SO saponins in THP-1 cell line. (**C**) Cytotoxicity of SO1406 in different cell lines. (**D**) Cytotoxicity of saporin in MDA-MB231 cell line. (**E**) Cytotoxicity of saporin in MDA-MB231 cell line in the presence of SO1406 at different concentrations.

Hemolytic activity of the natural saponins was measured in an in vitro assay on rabbit red blood cells. The Half-maximal Hemolytic Concentration (HC_50_) is the concentration of an agent (such as a saponin) required to induce 50% lysis of red blood cells. The HC_50_ values for SO1406, SO1448, SO1684, and SO1726 are 14.15, 12.36, 18.23, and 4.00 μM, respectively (Figure 4A).

### 3.3. Cytotoxicity

The intrinsic cytotoxicity of the isolated saponins was measured in THP-1 cells (Figure 4B). Among the four compounds, SO1406 exhibited the lowest cytotoxicity, with a CC_50_ value of 38.73 μM, whereas SO1726 displayed the highest cytotoxicity, with a CC_50_ of 2.87 μM. SO1448 and SO1684 showed intermediate CC_50_ values of 20.89 and 8.98 μM, respectively. Owing to its comparatively low toxicity and hemolytic activity, SO1406 was selected for further evaluation, and it exhibited consistent cytotoxicity across multiple human cancer cell lines, including THP-1 (acute monocytic leukemia), RPMI 8226 (multiple myeloma), MDA-MB231 (triple-negative breast cancer), HepG2 (hepatocellular carcinoma), and FaDu (hypopharyngeal squamous cell carcinoma) (Figure 4C).

### 3.4. Synergistic Effect

The CC_50_ of SAP in MDA-MB-231 cells was determined to be 0.80 μM (Figure 4D), while that of SO1406 alone was 11.32 μM (Figure 4C). Remarkably, in the presence of 1.33 μg/mL (0.95 μM) of SO1406, a concentration at which SO1406 alone was non-toxic, the CC_50_ of SAP decreased dramatically to 6.94 × 10^−5^ μM (Figure 4E).

## 4. Discussion

Two pairs of structurally defined pure saponins were obtained. SO1726 and SO1684 share the same core structure 3-*O-β*-D-Gal-(1→2)-[*β*-D-Xyl-(1→3)]-*β*-D-GlcA-gypsogenin-28-*O*-*β*-D-Qui-(1→4)-[*β*-D-Xyl-(1→3)-*β*-D-Xyl-(1→4)]-*α*-L-Rha-(1→2)-*β*-D-Fuc, with both SO1684 and SO1726 acetylated at Qui O-4 and SO1726 bearing additional acetylation at Qui O-3. Gypsogenin is a common aglycon in saponins from the Caryophyllaceae family [48]. However, earlier studies reported that saponins isolated from *S. officinalis* predominantly contain gypsogenic, 16-hydroxygypsogenic acid, or quillaic acid aglycons [49,50,51,52,53,54]. Only recently have root saponins from *S. officinalis* been identified with a gypsogenin aglycon [34,55]. Despite these investigations, the structural landscape of *S. officinalis* saponins remains incompletely defined.

The structure of SO1726 is strikingly similar to that of a saponin isolated from *Gypsophila perfoliata* (compound 3) [56]. The only structural difference lies in the terminal sugar residue of the C-28-linked pentasaccharide chain, which is a *β*-D-Xyl unit in SO1726, whereas it is an *α*-L-arabinopyranosyl unit in *G. perfoliate* 3. This distinction was confirmed by analysis of both ^1^H and ^13^C NMR spectra (Table S3).

SO1684, a known saponin, possesses the same structural backbone as SO1726, differing only by the presence of a single acetyl group at the Qui *O*-4 (Figure 2 for structure and Figure S1 for NMR comparisons). This structure is identical to that of saponins previously isolated from the roots of *Acanthophyllum laxiusculum* Schiman-Czeika [57] and from the roots of *Gypsophila arrostii var. nebulosa* and *Gypsophila bicolor* [58]. A closely related isomer, *glanduloside* D, isolated from the roots of *Acanthophyllum glandulosum*, bears the acetyl group at the Qui 3-O position rather than 4-O position [59]. Deacetylation of either SO1684 or SO1726 afforded another known saponin SA1641 isolated from Saponinum album (Merck). Similarly, SO1406 and SO1448 share the same core of 3-*O*-*β*-D-Gal-(1→2)-[*β*-D-Xyl-(1→3)]-*β*-D-GlcA-gypsogenin-28-*O*-*β*-D-Xyl-(1→4)-*α*-L-Rha-(1→2)-*β*-D-Fuc, and both have an acetyl group at Fuc O-4 but SO1448 contains an additional acetyl group at Fuc O-3.

With the purified saponins in hand, we evaluated their ability to enhance SAP cytotoxicity. SAP is a type I ribosome-inactivating protein (RIP-I). Unlike type II RIPs, which contain both an enzymatic A-chain and a B-chain that mediates cell binding and uptake, SAP comprises only the A-chain. As a result, it undergoes inefficient cellular internalization, endosomal escape, and cytosolic delivery to ribosomes. To improve SAP’s therapeutic efficacy, strategies such as the use of EEEs have been investigated [10,47].

Saponins are known for their hemolytic activity, which partially contributes to their toxicity and the tendency to induce tissue damage [60]. Hemolytic activity of the natural saponins was measured in an in vitro assay on rabbit red blood cells. Owing to its comparatively low toxicity (CC_50_ = 11.32 μM in MDA-MB-231 cells) and hemolytic activity (HC_50_ = 14.15 μM), SO1406 was selected for further evaluation. In the presence of SO1406 at 5 μg/mL, SAP CC_50_ values were significantly reduced in MDA-MB231, HepG2, FaDu, and RPMI 8266 cells, with the greatest cytotoxic enhancement observed in MDA-MB231 cells. Subsequent studies therefore focused on optimizing this synergy in this cell line. In the presence of 1.33 μg/mL (0.95 μM) of SO1406, a concentration at which SO1406 alone was non-toxic, the CC_50_ of SAP decreased dramatically from 0.80 μM (Figure 4D) to 6.94 × 10^−5^ μM in MDA-MB-231 cells (Figure 4E), representing an approximately 1.63 × 10^5^-fold decrease in SAP’s CC_50_ (For synergy analysis, see SI). These results demonstrate that SO1406 can substantially potentiate the efficacy of SAP, thereby enabling potent anticancer activity at markedly lower and potentially safer toxin doses.

The remarkable enhancement of SAP’s cytotoxicity by SO1406 suggests that this saponin could facilitate intracellular delivery of SAP to the cytosol, where ribosomes reside, a mechanism previously proposed for certain saponin-based EEEs. The low intrinsic cytotoxicity of SO1406, combined with its potent synergistic effect on SAP activity, underscores its potential as a promising adjuvant for protein toxin-based therapeutics. The results in Figure 4A,B demonstrate structure-dependent biological activities; however, currently available data is not sufficient to elucidate a reliable structure–activity relationship.

## 5. Conclusions

We successfully isolated and characterized four structurally defined saponins, SO1684, SO1726, SO1406, and SO1448, from the roots of *Saponaria officinalis*. Among these, SO1406 demonstrated a remarkable ability to enhance the cytotoxicity of the ribosome-inactivating SAP by several orders of magnitude in vitro. These findings not only highlight the potential of SO1406 as a powerful cytotoxicity enhancing agent but also reinforce the broader promise of structurally defined natural saponins in improving the intracellular delivery and therapeutic efficacy of protein-based anticancer agents. Further studies focused on mechanistic elucidation, SAR optimization, and in vivo validation will be critical for advancing SO1406 and related saponins toward clinical application.

## 6. Patents

A patent application partially based on this work has been filed.

## Figures and Tables

**Table 1:** ^1^H and ^13^C NMR data (δ, ppm) of the sugar moieties of SO1726 (C_5_D_5_N, 850 MHz) and SO1406 (CD_3_OD, 850 MHz).

	SO1726 (C)		SO1406 (D)	
3-*O*-	^13^C	^1^H	^13^C	^1^H
GlcA	δ_c_	δ_H_ (m, *J* (Hz))	δ_c_	δ_H_ (m, *J* (Hz))
1	104.53	4.87 (*d*, 7.1)	104.58	4.46 (*d*, 7.4)
2	78.66	4.32 (*m*)	78.11	3.66 (*d*, 8.9)
3	86.58	4.27 (*m*)	86.61	3.69 (*d*, 7.6)
4	71.86	4.45 (*m*)	71.39	3.56 (*m*)
5	77.76	4.49 (*m*)	76.52	3.81 (*m*)
6	nd	-	170.31	-
Gal				
1	104.48	5.59 (*d*, 7.5)	103.76	4.80 (*d*, 7.0)
2	73.99	4.51 (*m*)	73.58	3.44 (*m*)
3	75.94	4.16 (*m*)	75.36	3.43 (*m*)
4	70.65	4.59 (*m*)	70.77	3.83 (*m*)
5	76.95	4.01 (*m*)	76.66	3.49 (*m*)
6	61.99	4.41 (*m*)	62.15	3.76 (*m*)
		4.55 (*m*)		3.75 (*m*)
Xyl (I)				
1	105.5	5.36 (*d*, 7.5)	104.93	4.57 (*d*, 7.7)
2	75.78	3.97 (*m*)	75.24	3.23 (*d*, 9.1)
3	79.08	4.13 (*m*)	78.21	3.31 (*m*)
4	71.29	4.15 (*m*)	70.93	3.49 (*m*)
5	67.73	3.68 (*m*)	67.15	3.90 (*m*)
		4.28 (*m*)		3.25 (*t*, 10.6)
28-*O*-				
Fuc				
1	94.79	5.97 (*d*, 8.2)	94.99	5.40 (*d*, 8.1)
2	74.62	4.56 (*m*)	75.07	3.76 (*m*)
3	77.12	4.21 (*m*)	74.79	3.89 (*m*)
4	84.28	3.99 (*m*)	75.23	5.08 (*d*, 3.5)
5	71.78	3.93 (*q*, 6.6)	71.13	3.85 (*m*)
6	17.49	1.51 (*d*, 6.3)	16.49	1.07 (*d*, 6.4)
Qui				
1	106.23	5.07 (*d*, 7.8)		
2	73.58	4.04 (*m*)		
3	76.63	5.64 (*t*, 9.5)		
4	74.8	5.08 (*s*)		
5	70.71	3.71 (*m*)		
6	18.16	1.26 (*d*, 6.1)		
Rha				
1	101.84	6.32 (s, br)	101.64	5.34 (*d*, 1.4)
2	72.01	4.75 (s, br)	71.87	3.94 (*dd*, 3.0, 1.8)
3	72.94	4.59 (*m*)	72.29	3.81 (*m*)
4	85.78	4.31 (*m*)	85.01	3.49 (*m*)
5	68.49	4.39 (*m*)	68.81	3.80 (*m*)
6	18.95	1.65 (*d*, 6.1)	18.29	1.28 (*d*, 6.2)
Xyl (II)				
1	107.46	5.02 (*d*, 7.4)	107.48	4.41 (*d*, 7.7)
2	75.47	3.99 (*m*)	76.26	3.17 (*t*, 9.5)
3	87.58	4.03 (*m)*	78.35	3.30 (*m*)
4	69.24	4.11 (*m*)	70.99	3.47 (*m*)
5	67.45	3.5 (*t*, 10.8)	67.26	3.83 (*m*)
		4.24 (*m*)		3.16 (*t*, 9.8)
Xyl (III)				
1	106.17	5.19 (*d*, 7.5)		
2	75.47	4.07 (*m*)		
3	78.46	4.15 (*m*)		
4	71.18	4.15 (*m*)		
5	67.83	4.29 (*m*)		
		3.67 (*m*)		
Qui-3-OAc	170.83			
	21.18	1.98 (*s*)		
Qui-4-OAc	170.56			
	21.16	2.06 (*s*)		
Fuc-4-OAc			172.78	
			20.80	2.16 (*s*)

## Data Availability

The original contributions presented in this study are included in the article. Further inquiries can be directed to the corresponding author.

## Supplementary Materials

The following supporting information can be downloaded at https://www.mdpi.com/article/10.3390/biomedicines14030626/s1, Table S1. Comparison of ^13^C NMR of SA1641 and deacetylated SO1726; Table S2. Comparison of characteristic ^1^H NMR peaks of SA1641 and deacetylated SO1726 and SO1684; Table S3. Comparison of characteristic ^1^H and ^13^C NMR peaks of *Gypsophila perfoliate* (compound 3) and SO1726; Figure S1. Proton NMR of SO1684 and SO1726 show downfield shifts of quinovosyl protons affected by acetylation; Figure S2. Proton NMR of SO1448 and SO1406 show downfield shifts of fucosyl protons affected by acetylation; 1D Proton and carbon NMR spectra of SO1406, SO1448, SO1684, SO1726; 2D NMR spectra of SO1406 and SO1726; HRMS spectra of SO1406, SO1448, SO1684, SO1726; Data of synergy analysis.

## Abbreviations

The following abbreviations are used in this manuscript:

ATCC	American Type Culture Collection
ATP	Adenosine triphosphate
COSY	Homonuclear correlated spectroscopy
ESI–TOF	Electrospray ionization time-of-flight mass spectrometry
HMBC	Heteronuclear multiple-bond correlation spectroscopy
HSQC	Heteronuclear single quantum coherence
NOESY	Nuclear Overhauser effect spectroscopy
NMR	Nuclear magnetic resonance
PBS	Phosphate-buffered saline
RBC	Red blood cell
TOCSY	Total correlation spectroscopy
