# Supplementary material for: Improving Cytotoxicity of Saporin with Saponin SO1406 Isolated from the Roots of Saponaria Officinalis
Source: Biomedicines. 2026 Mar 11;14(3):626. doi: 10.3390/biomedicines14030626 (PMC13023535; doi:10.3390/biomedicines14030626)

Improving cytotoxicity of saporin with saponin SO1406 isolated from the roots of *Saponaria officinalis*

Chaeeun Lim-Paik,[1] Qinghua Zeng,[2] Rebekah Beyea,[3] Rebecca Boohaker,[2] and Pengfei Wang*[1]

[1]Department of Chemistry, University of Alabama at Birmingham, 901 14th Street South, Birmingham, AL 35294, U.S.A. [2]Southern Research Institute, 2000 9th Avenue South, Birmingham, AL 35205, U.S.A. [3]Adjuvax LLC, 2000 9th Avenue South, Birmingham, AL35205, U.S.A.

wangp@uab.edu

Table of Contents

	Page number
**Table S1**. Comparison of ^{13}C NMR of SA1641 and deacetylated SO1726 and SO1684.	S2
**Table S2**. Comparison of characteristic ^{1}H NMR peaks of SA1641 and deacetylated SO1726 and SO1684.	S3
**Table S3**. Comparison of characteristic ^{1}H and ^{13}C NMR peaks of *Gypsophila perfoliate* (compound 3) and SO1726.	S4
**Figure S1**. Proton NMR of SO1684 and SO1726 show downfield shifts of quinovosyl protons affected by acetylation.	S5
**Figure S2**. Proton NMR of SO1448 and SO1406 show downfield shifts of fucosyl protons affected by acetylation.	S6
^{1}H NMR of SO1406	S7
^{13}C NMR of SO1406	S8
^{1}H NMR of SO1448	S9
^{13}C NMR of SO1448	S10
^{1}H NMR of SO1684	S11
^{13}C NMR of SO1684	S12
^{1}H NMR of SO1726	S13
^{13}C NMR of SO1726	S14
2D NMR of SO1406	S15-22
2D NMR of SO1726	S23-30
HRMS of SO1726, 1684, 1448, and 1406	S31-34
Synergy analysis	S35

Table S1. Comparison of ^{13}C NMR of SA1641 and deacetylated SO1726 and SO1684 (C_5D_5N, 214 MHz).[a, b, c]

	Gypsogenin				3-O-sugars				28-O-sugars	
	SA1641	Deacetylated SO1726/SO1684			SA1641	Deacetylated SO1726/SO1684			SA1641	Deacetylated SO1726/SO1684
1	38.0	37.8		GlcUA				Rha		
2	25.0	25.2		1	103.6	103.86		1	101.1	101.1
3	85.1	85.2		2	77.8	77.9		2	71.4	71.4
4	55.0	54.8		3	85.9	85.9		3	72.5	72.3
5	48.2	47.6		4	71.0	71.1		4	84.8	84.8
6	20.5	20.5		5	77.0	77.1		5	68.0	67.8
7	32.5	32.4		6	n.d.	n.d.		6	18.2	18.4
8	40.1	39.9		Gal				Fuc		
9	47.5	47.6		1	103.8	103.9		1	94.1	94.2
10	36.0	36.0		2	73.4	73.3		2	74.1	74.1
11	23.4	23.4		3	75.2	75.3		3	76.7	76.8
12	122.2	122.2		4	69.9	69.9		4	84.0	84.0
13	143.5	143.8		5	76.3	76.4		5	71.3	71.3
14	41.9	42.0		6	61.3	61.3		6	18.2	18.3
15	28.1	28.3		Xyl (I)				Xyl (II)		
16	23.7	23.4		1	104.7	104.8		1	106.6	106.8
17	48.3	48.8		2	74.8	74.8		2	75.0	75.1
18	41.9	41.8		3	78.2	78.4		3	86.8	86.8
19	46.0	46.0		4	70.5	70.6		4	68.2	68.6
20	30.5	30.4		5	67.0	67.1		5	66.6	66.8
21	33.0	33.7						Xyl (III)		
22	32.2	32.0						1	105.3	105.5
23	211.8	210.9						2	75.1	75.2
24	10.8	11.0						3	77.7	77.8
25	15.2	15.5						4	70.4	70.5
26	17.1	17.2						5	66.9	67.0
27	25.7	25.6						Qui		
28	177.0	176.3						1	106.2	106.5
29	32.8	32.9						2	75.5	75.6
30	23.0	22.9						3	78.1	78.2
								4	76.1	76.3
								5	73.0	73.0
								6	16.9	16.9

[a] Chemical shift in ppm. [b] Deacetylated SO1726 and SO1684 are identical. [c] Data of SA1641 is from Ref. 17: Weng, A., et al., *The toxin component of targeted anti-tumor toxins determines their efficacy increase by saponins.* Mol Oncol, 2012. **6**(3): p. 323-32.

Table S2. Comparison of characteristic ^{1}H NMR peaks of SA1641 and deacetylated SO1726 and SO1684 (C_5D_5N, 300 MHz).[a, b, c]

	SA1641	deacetylated SO1726/SO1684		SA1641	deacetylated SO1726/SO1684
3-O-trisaccharide			Gypsogenin		
GlcA H1	4.86	4.86	C-12	5.35	5.36
Gal H1	5.55	5.58	C-18	3.08	3.07
Xyl (I) H1	5.32	5.34	C-23	10.01	10.02
28-O-hexasaccharide			C-24	1.42	1.42
Rha H1	6.4	6.44	C-25	0.77	0.74
Rha H2	4.77	4.78	C-26	1	0.99
Fuc H1	5.95	5.96	C-27	1.18	1.16
Xyl (II) H1	5.01	5.02	C-28	-	-
Xyl (III) H1	5.17	5.18	C-29	0.84	0.82
Qui H1	4.96	4.98	C-30	0.81	0.79

[a] Chemical shift in ppm. [b] Deacetylated SO1726 and SO1684 are identical. [c] Data of SA1641 is from Ref. 17: Weng, A., et al., *The toxin component of targeted anti-tumor toxins determines their efficacy increase by saponins*. Mol Oncol, 2012. **6**(3): p. 323-32.

Table S3. Comparison of characteristic ^{1}H and ^{13}C NMR peaks of *G. perfoliate* 3 and SO1726 (C_5D_5N, 850 and 214 MHz).[a]

	Gypsophila perfoliate 3[b]		SO1726	
	^{1}H	^{13}C	^{1}H	^{13}C
28-O-				
Ara				
1	5.28	105.0		
2	4.73	71.4		
3	4.42	72.9		
4	4.31	69.3		
5	3.45, 4.20	66.9		
Xyl (III)				
1			5.19	106.17
2			4.07	75.47
3			4.15	78.46
4			4.15	71.18
5			3.67, 4.29	67.83

[a] Chemical shift in ppm. [b] Data from ref. 45: Chen, Q., J.-G. Luo, and L.-Y. Kong, New triterpenoid saponins from the roots of Gypsophila perfoliata Linn. Carbohydrate Research, 2011. 346: p. 2206-2212.

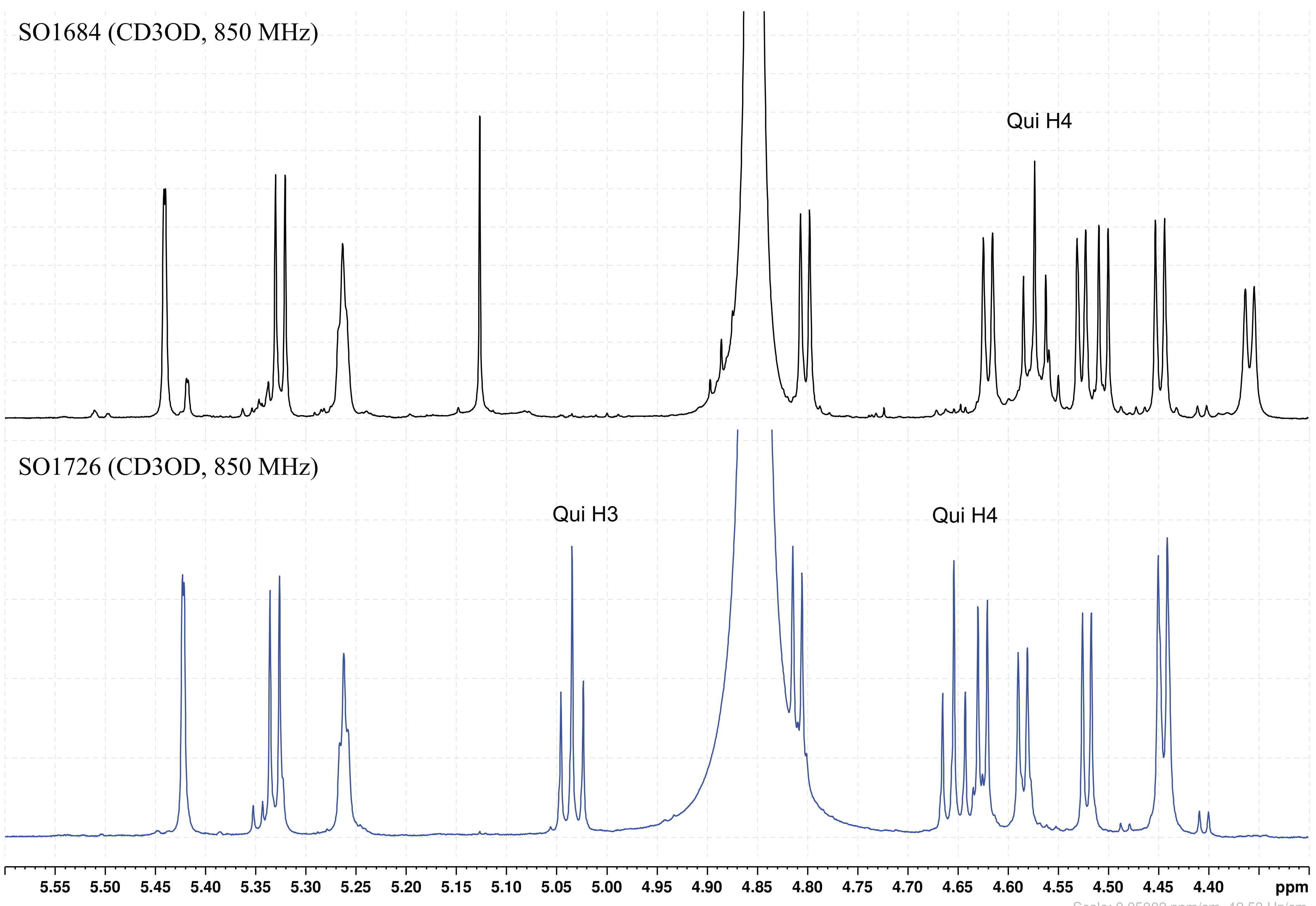

Figure S1. Proton NMR of SO1684 and SO1726 show downfield shifts of quinovosyl protons affected by acetylation.

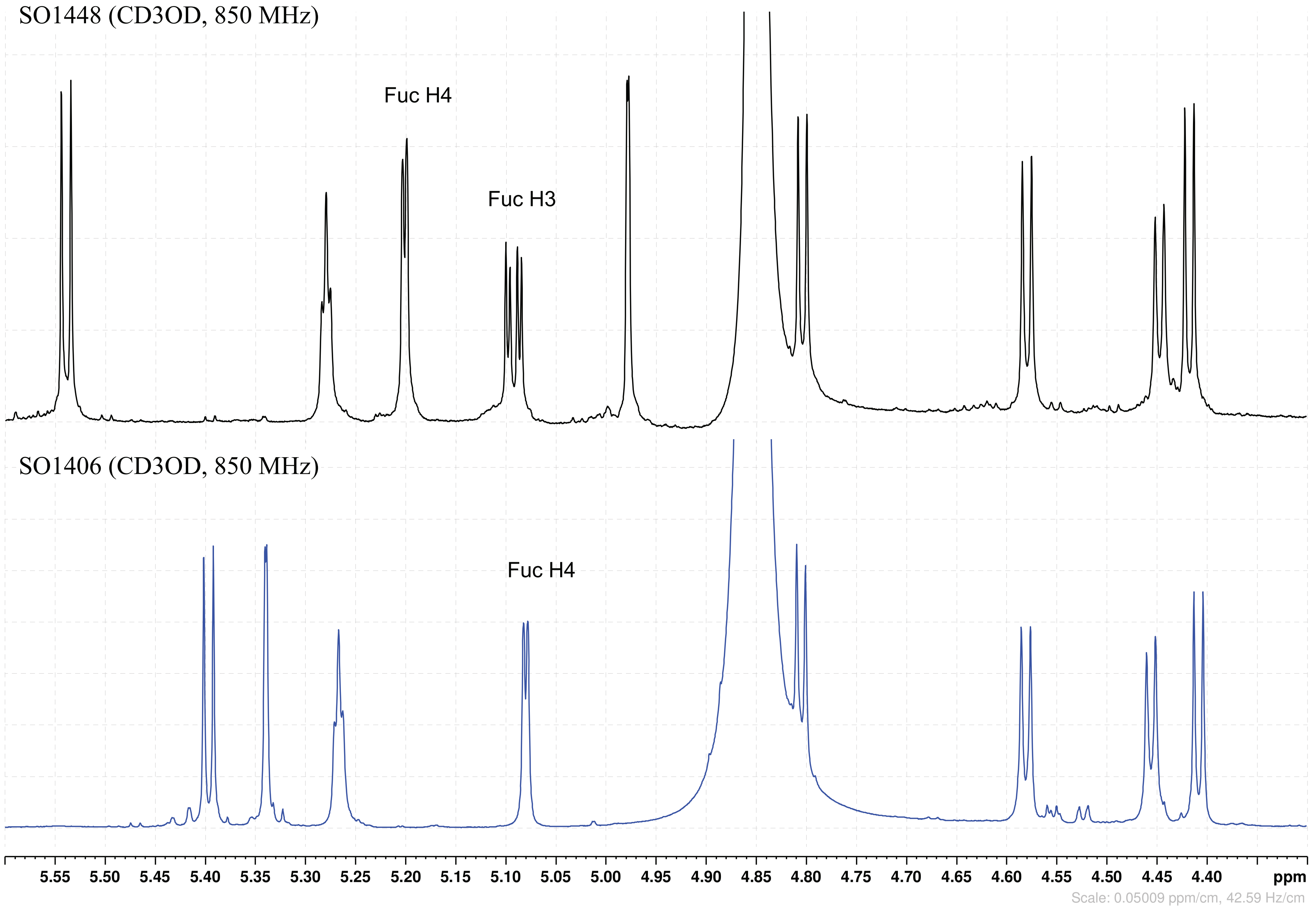

Figure S2 Proton NMR of SO1448 and SO1406 show downfield shifts of fucosyl protons affected by acetylation.

SO1406 (CD3OD, 850 MHz)

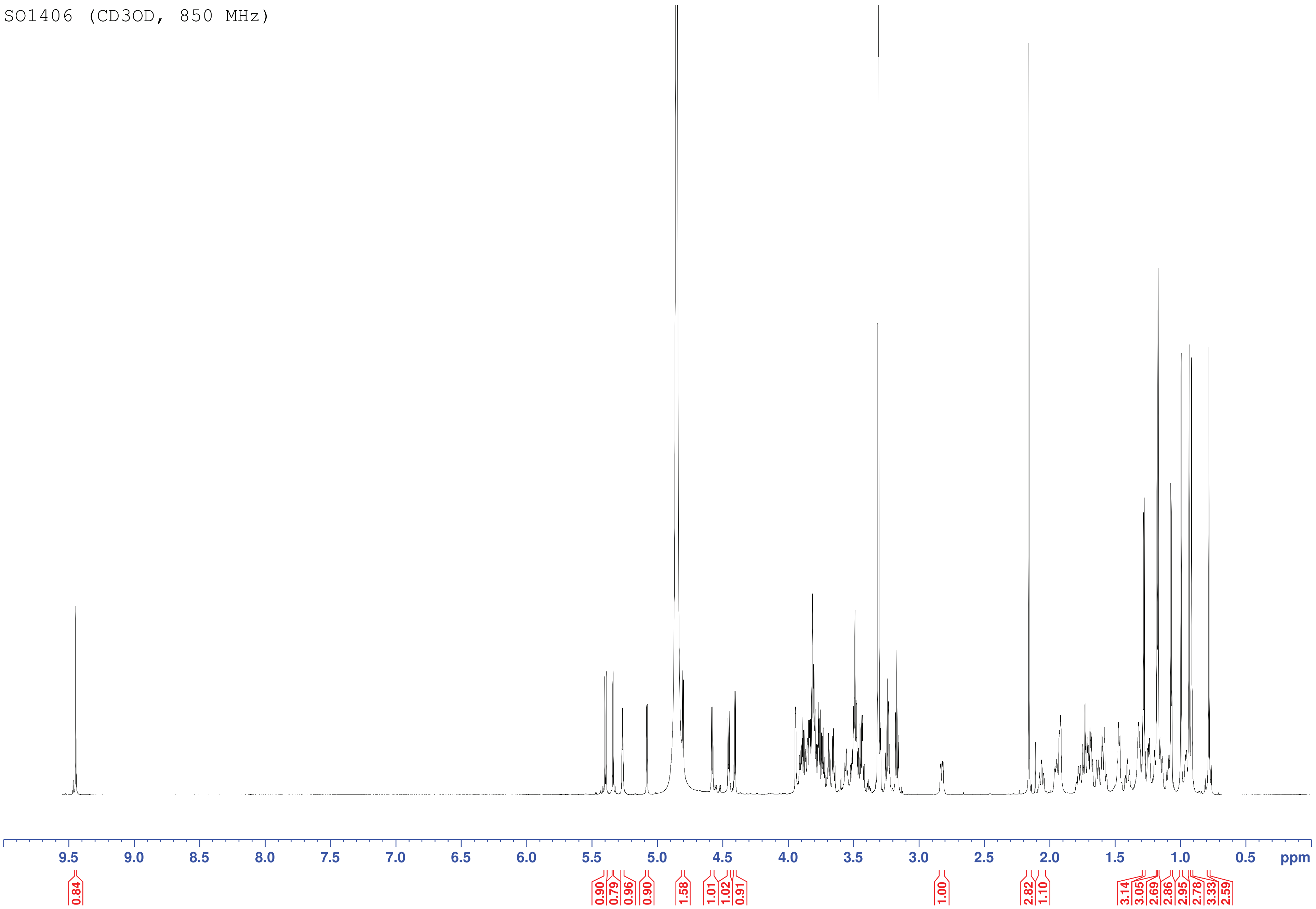

SO1406 (CD3OD, 214 MHz)

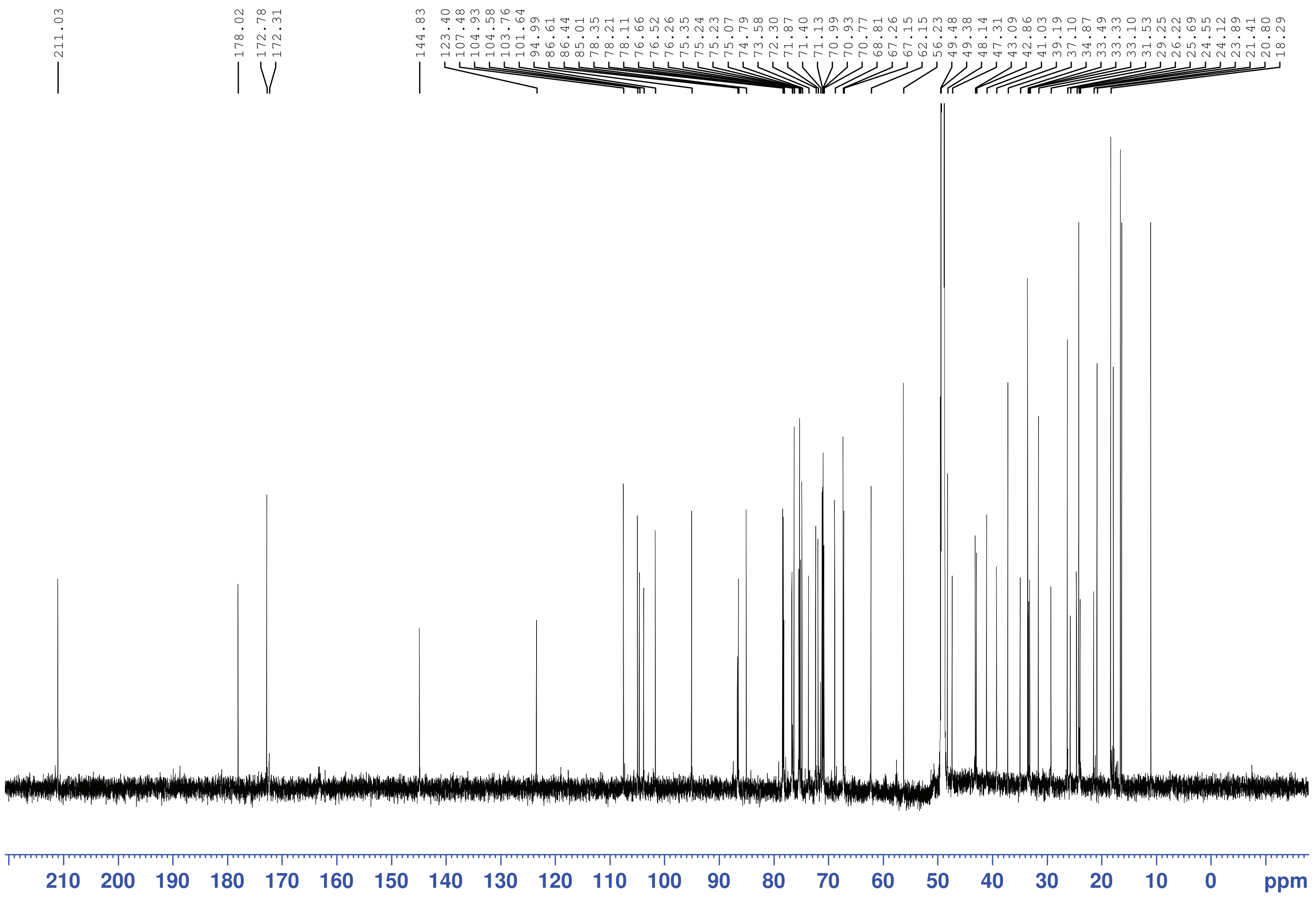

SO1448 (CD3OD, 850 MHz)

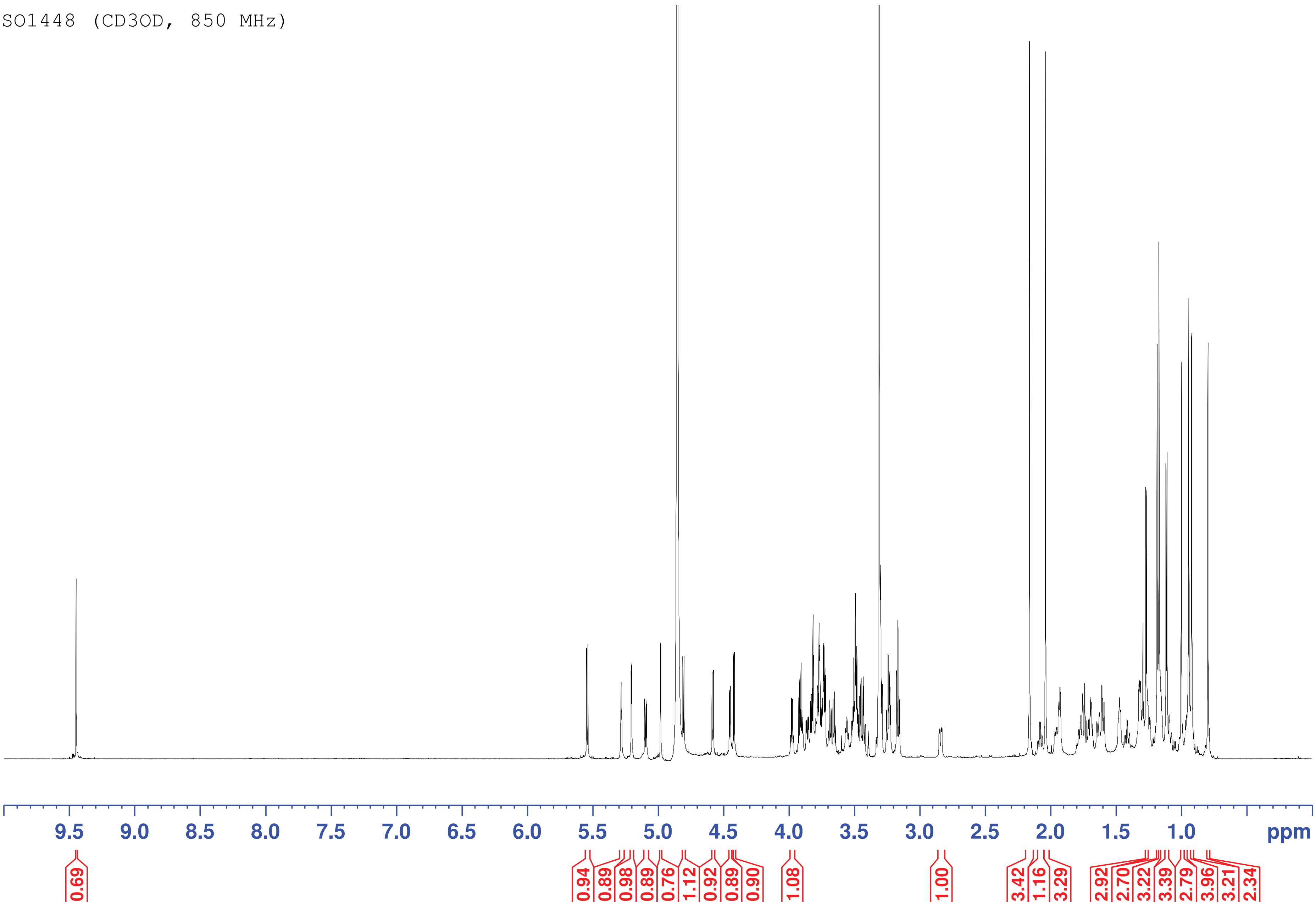

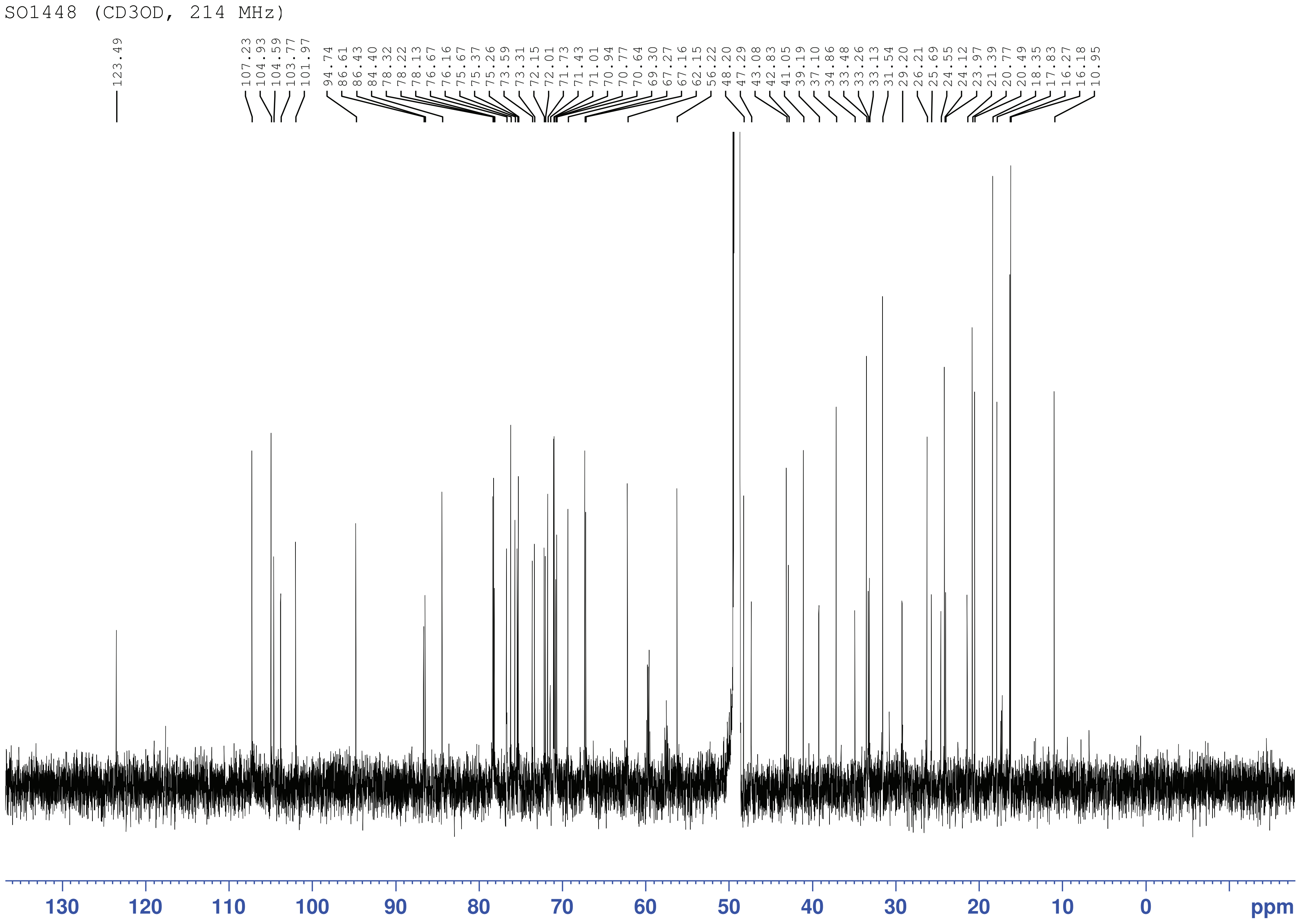
SO1448 (CD3OD, 214 MHz)
123.49
107.23
104.93
104.59
103.77
101.97
94.74
86.61
86.43
84.40
78.32
78.22
78.13
76.67
76.16
75.67
75.37
75.26
73.59
73.31
72.15
72.01
71.73
71.43
71.01
70.94
70.77
70.64
69.30
67.27
67.16
62.15
56.22
48.20
47.29
43.08
42.83
41.05
39.19
37.10
34.86
33.48
33.26
33.13
31.54
29.20
26.21
25.69
24.55
24.12
23.97
21.39
20.77
20.49
18.35
17.83
16.27
16.18
10.95
130
120
110
100
90
80
70
60
50
40
30
20
10
0
ppm

SO1684 (CD3OD, 850 MHz)

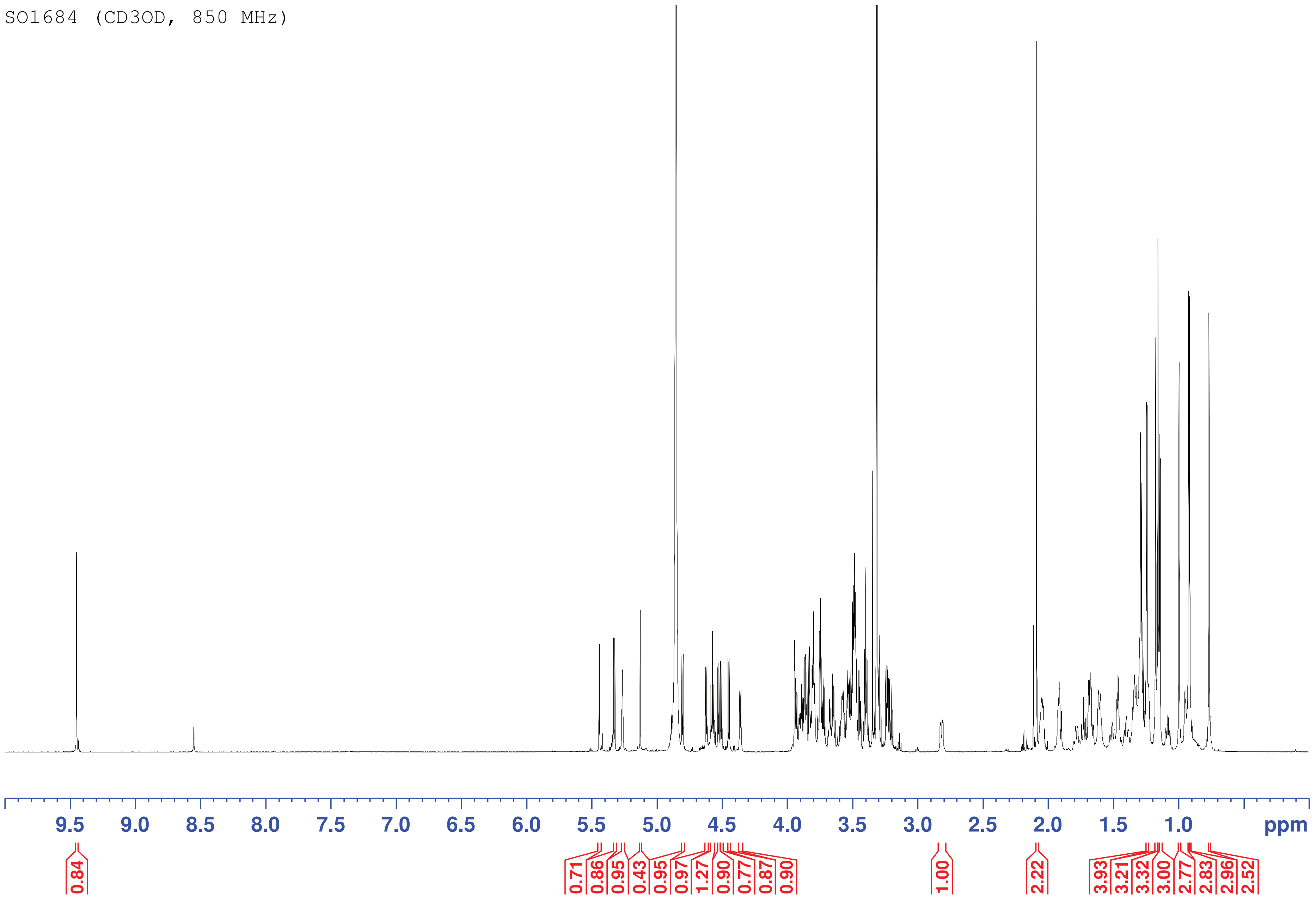

SO1684 (CD3OD, 214 MHz)

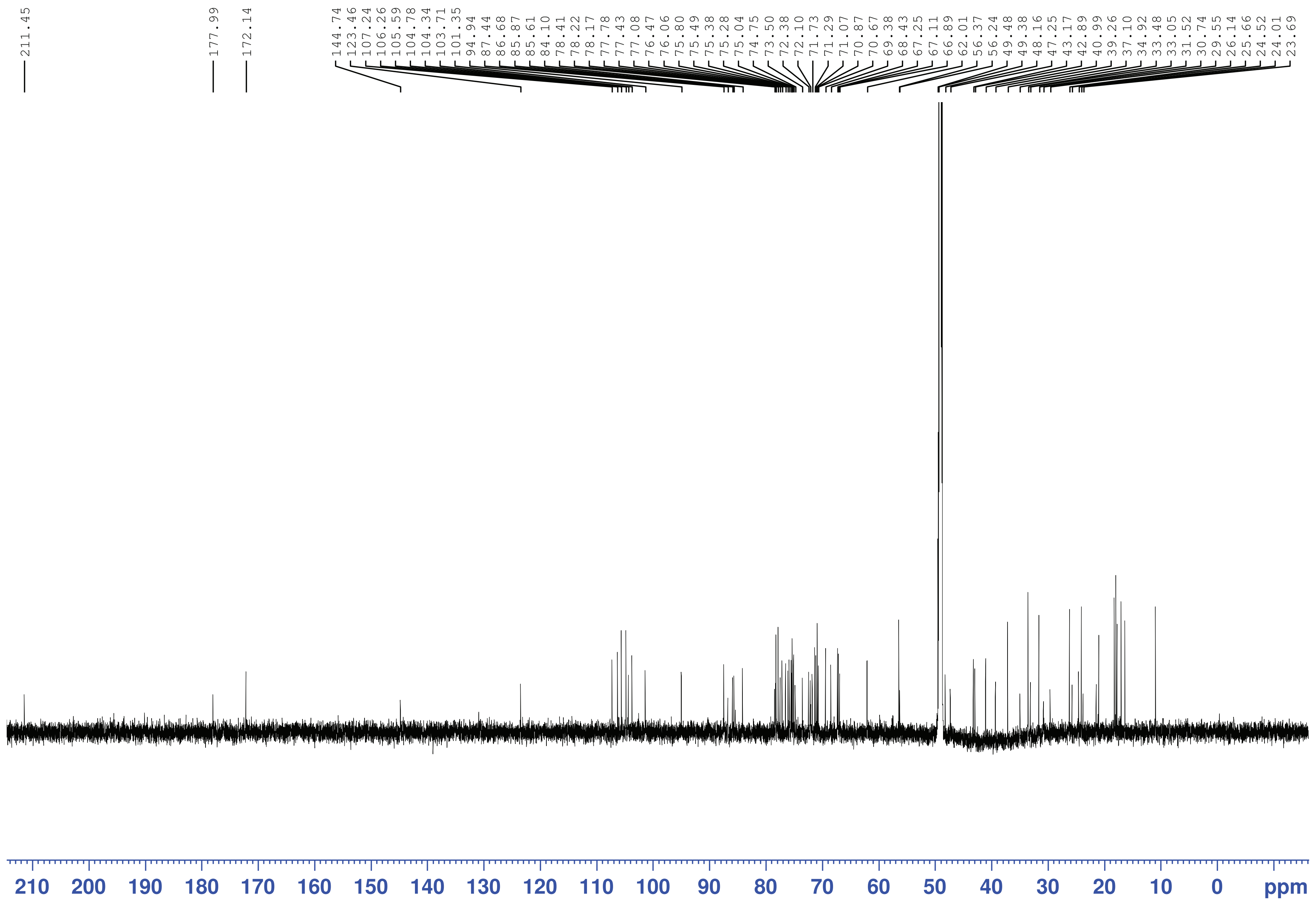

SO1726 (CD3OD, 850 MHz)

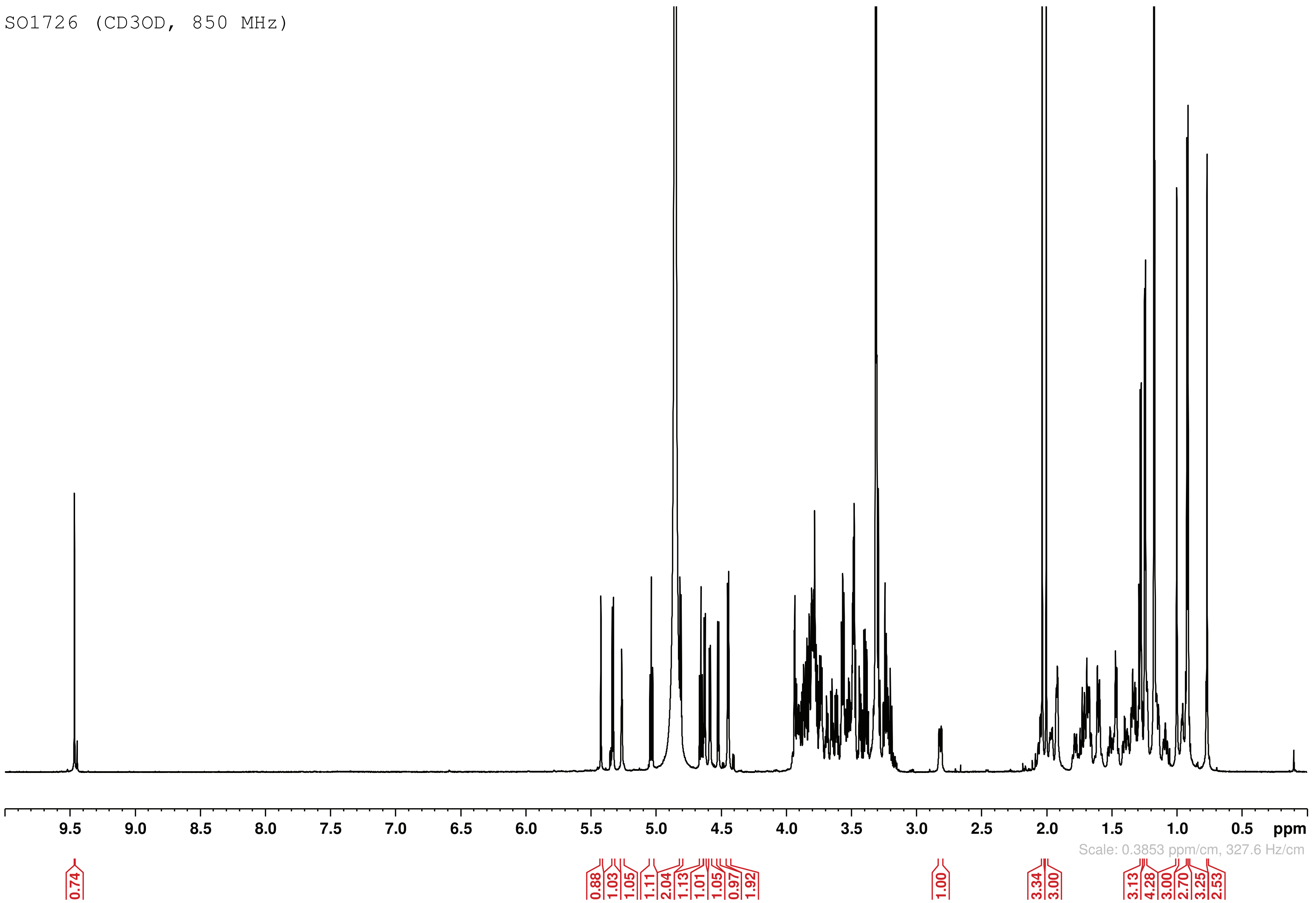

SO1726 (CD3OD, 214 MHz)

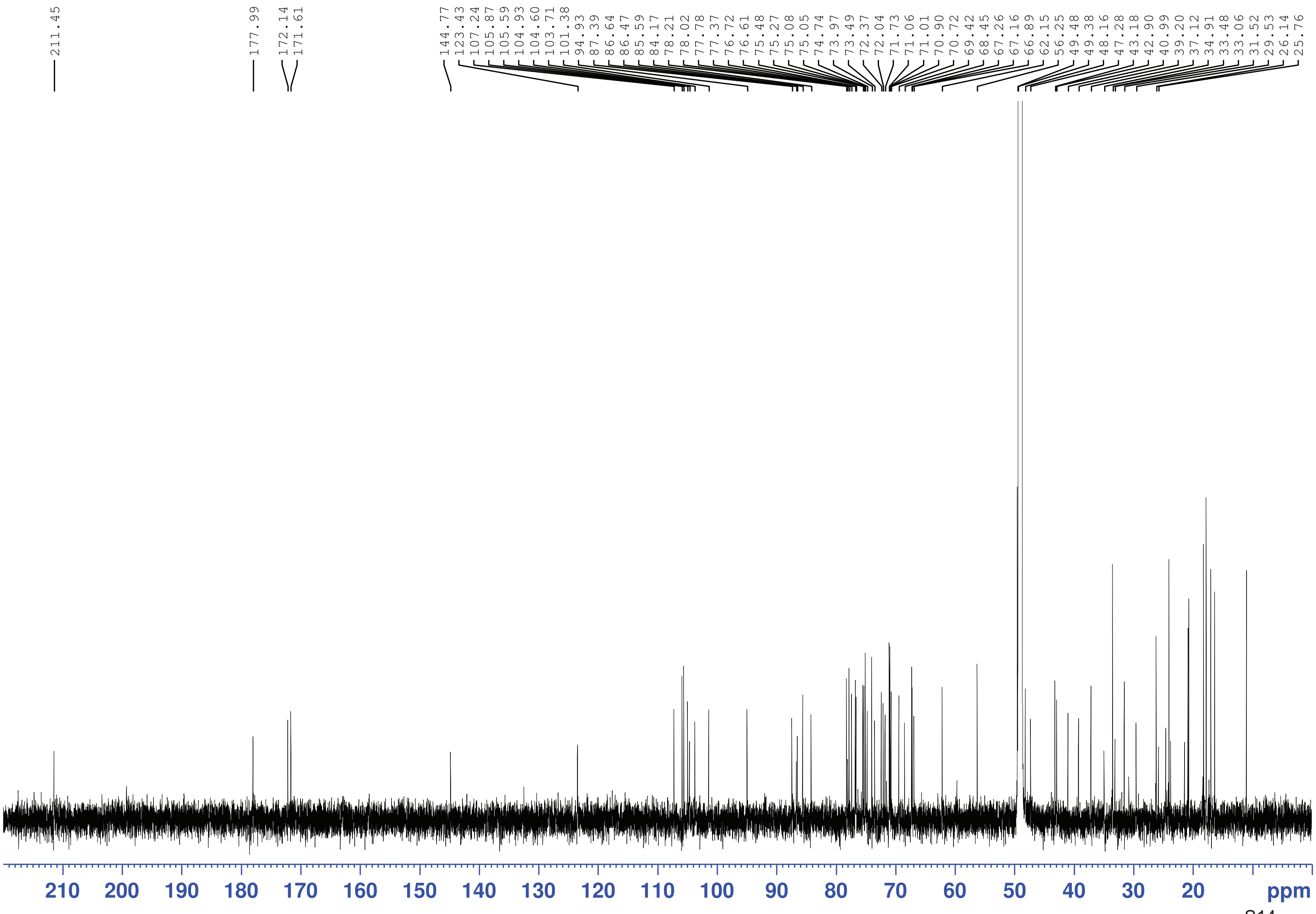

S14

SO1406 COSY (CD3OD, 600 MHz)

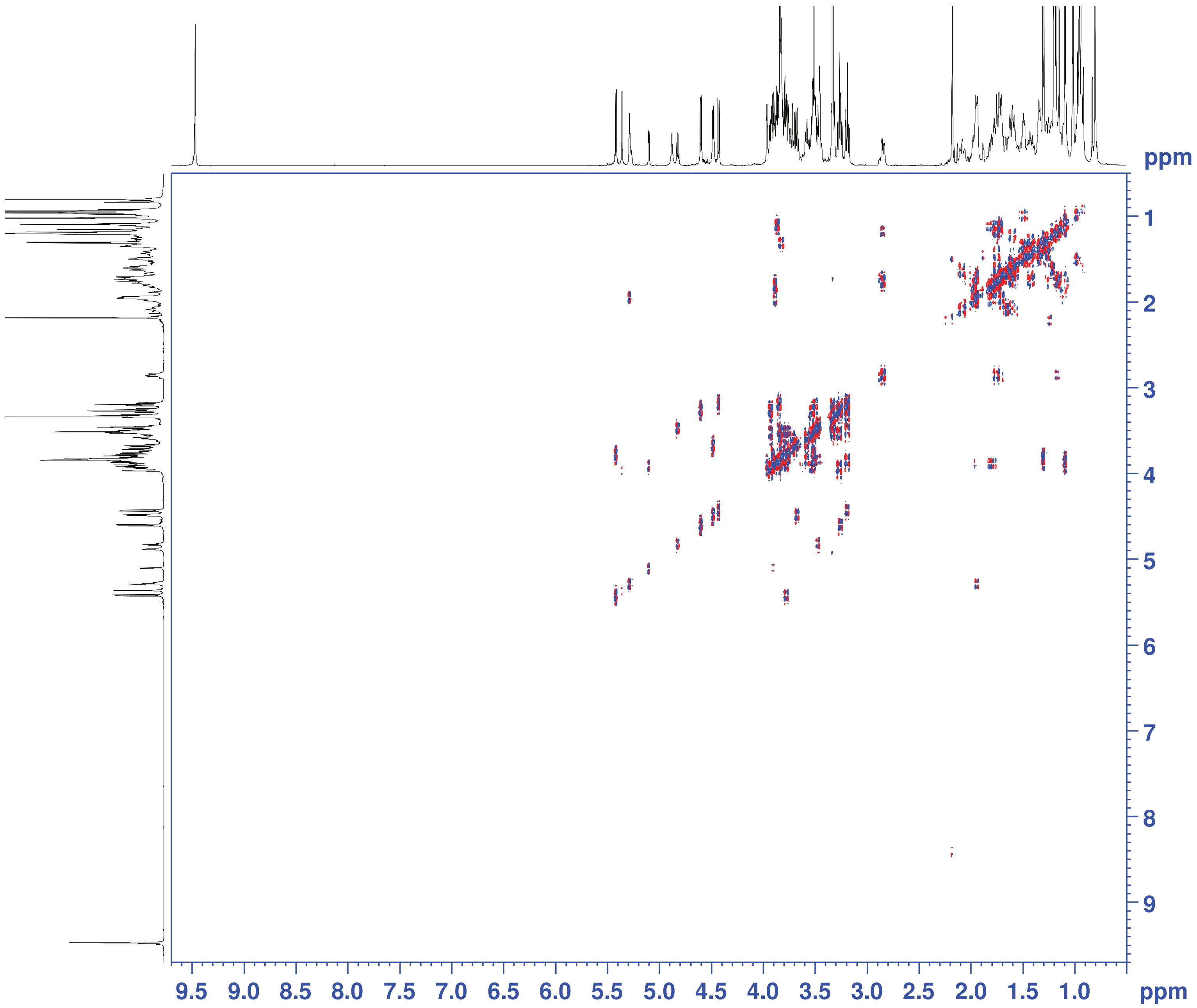

SO1406 COSY (CD3OD, 600 MHz)

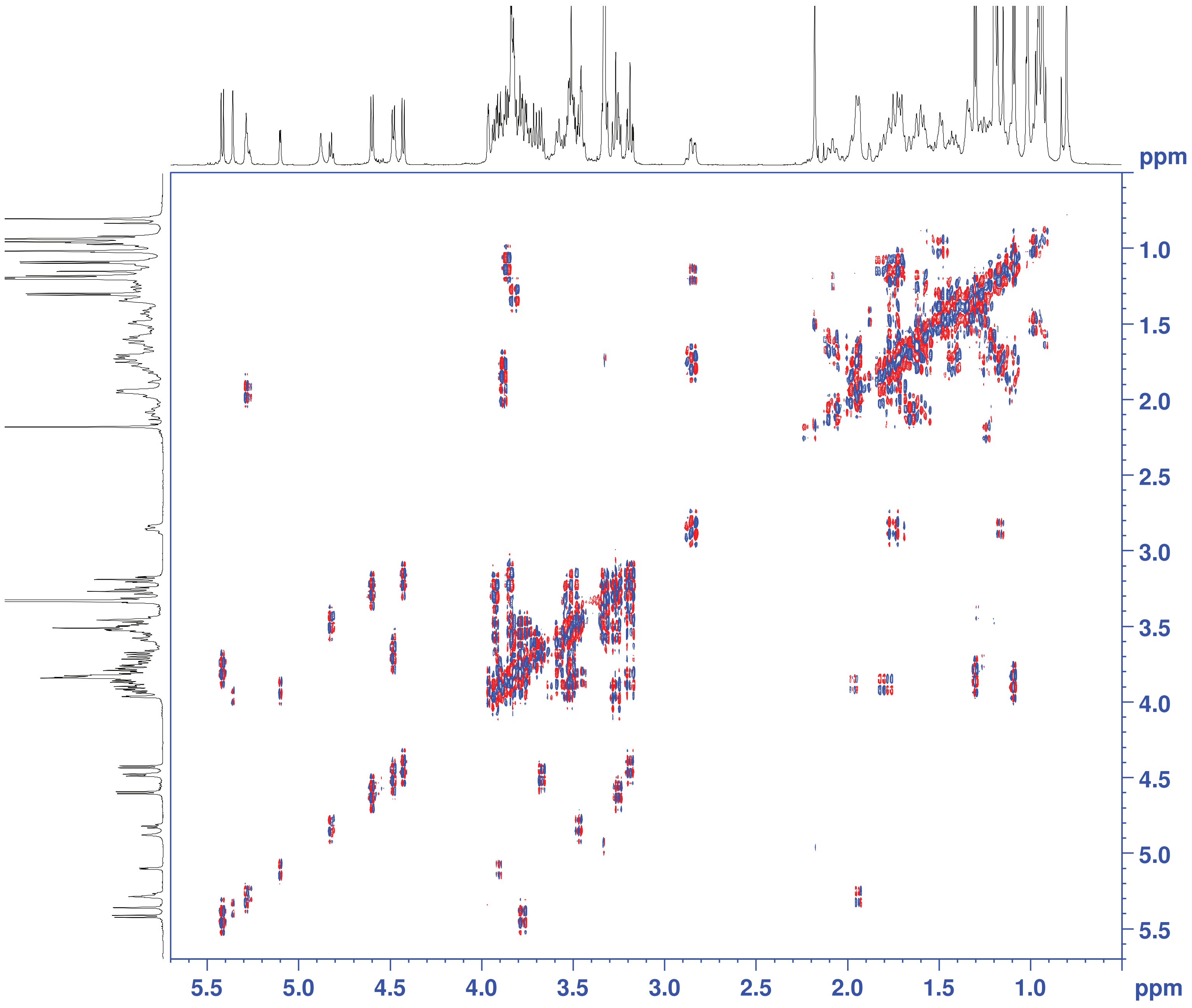

SO1406 TOCSY (CD3OD, 600 MHz)

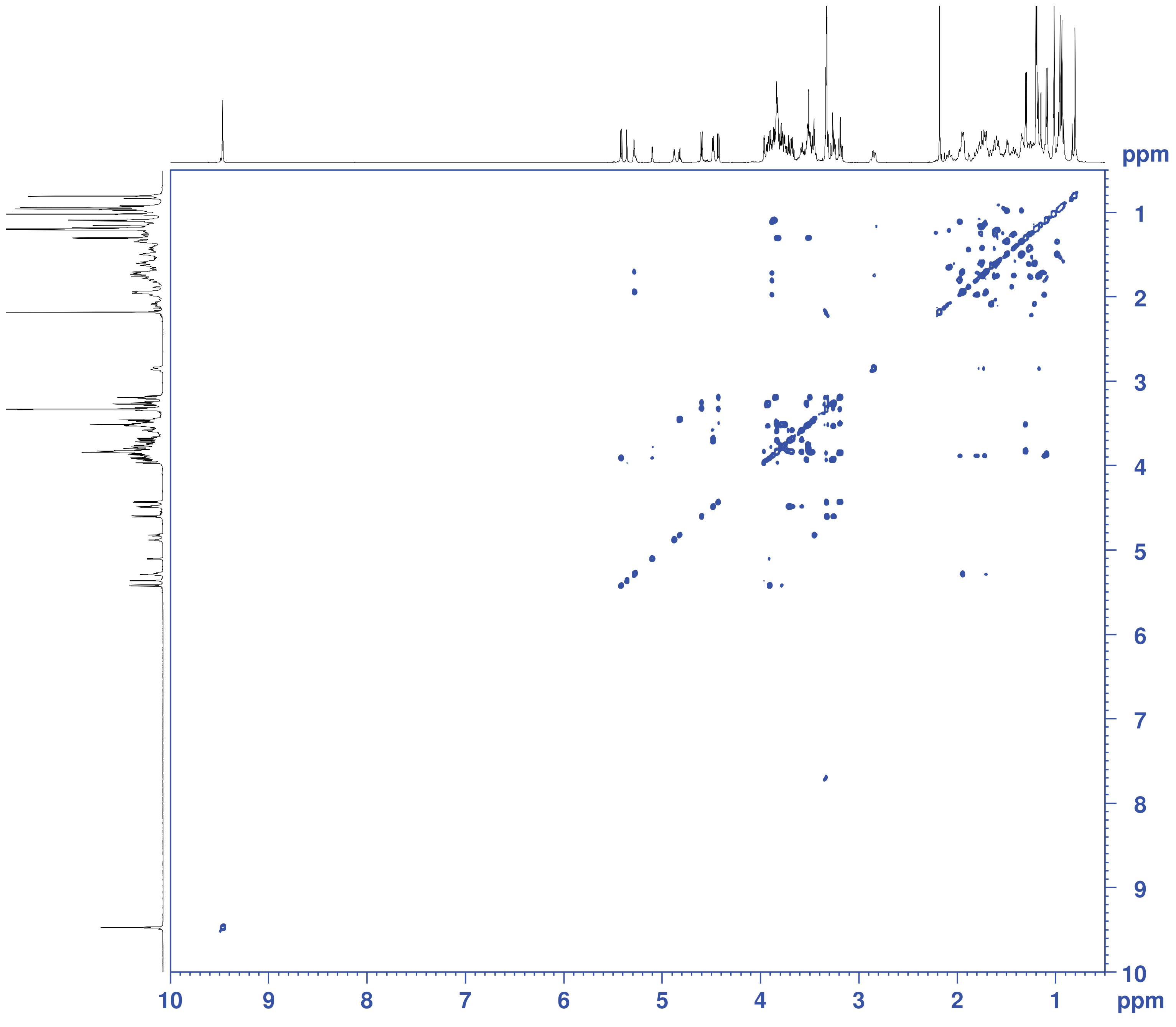

SO1406 TOCSY (CD3OD, 600 MHz)

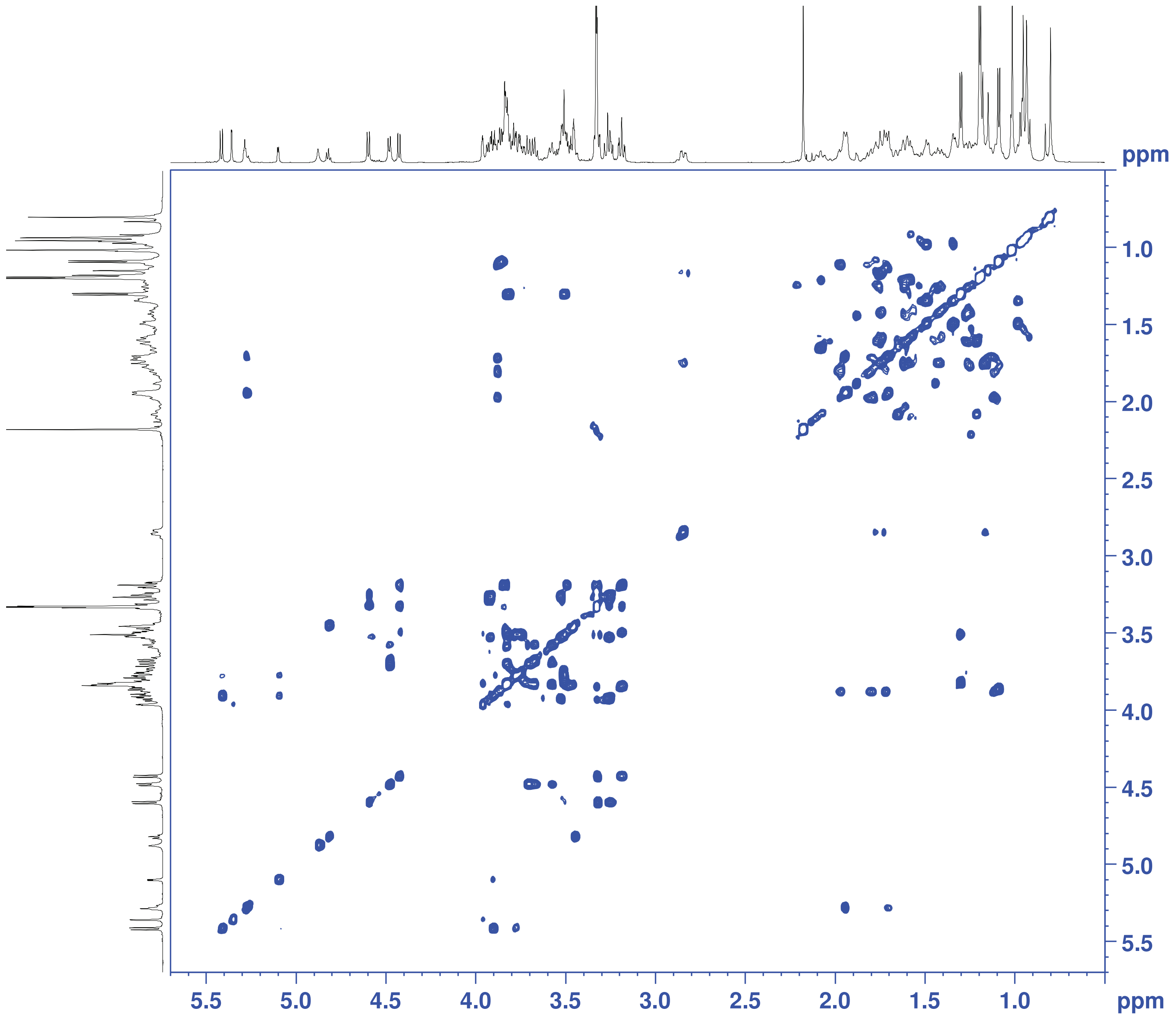

SO1406 HMBC (CD3OD, 600 MHz, 150 MHz)

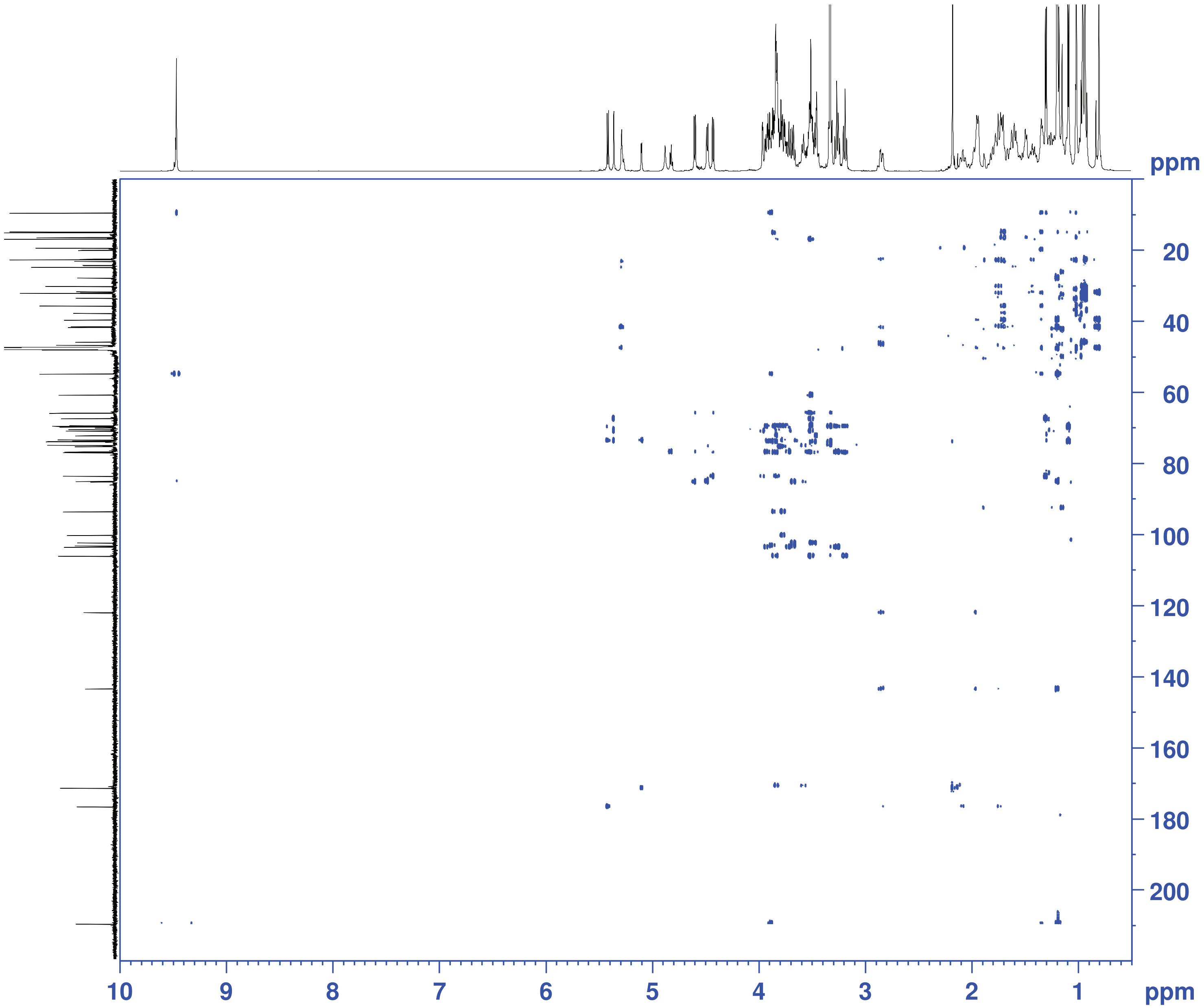

SO1406 HMBC (CD3OD, 600 MHz, 150 MHz)

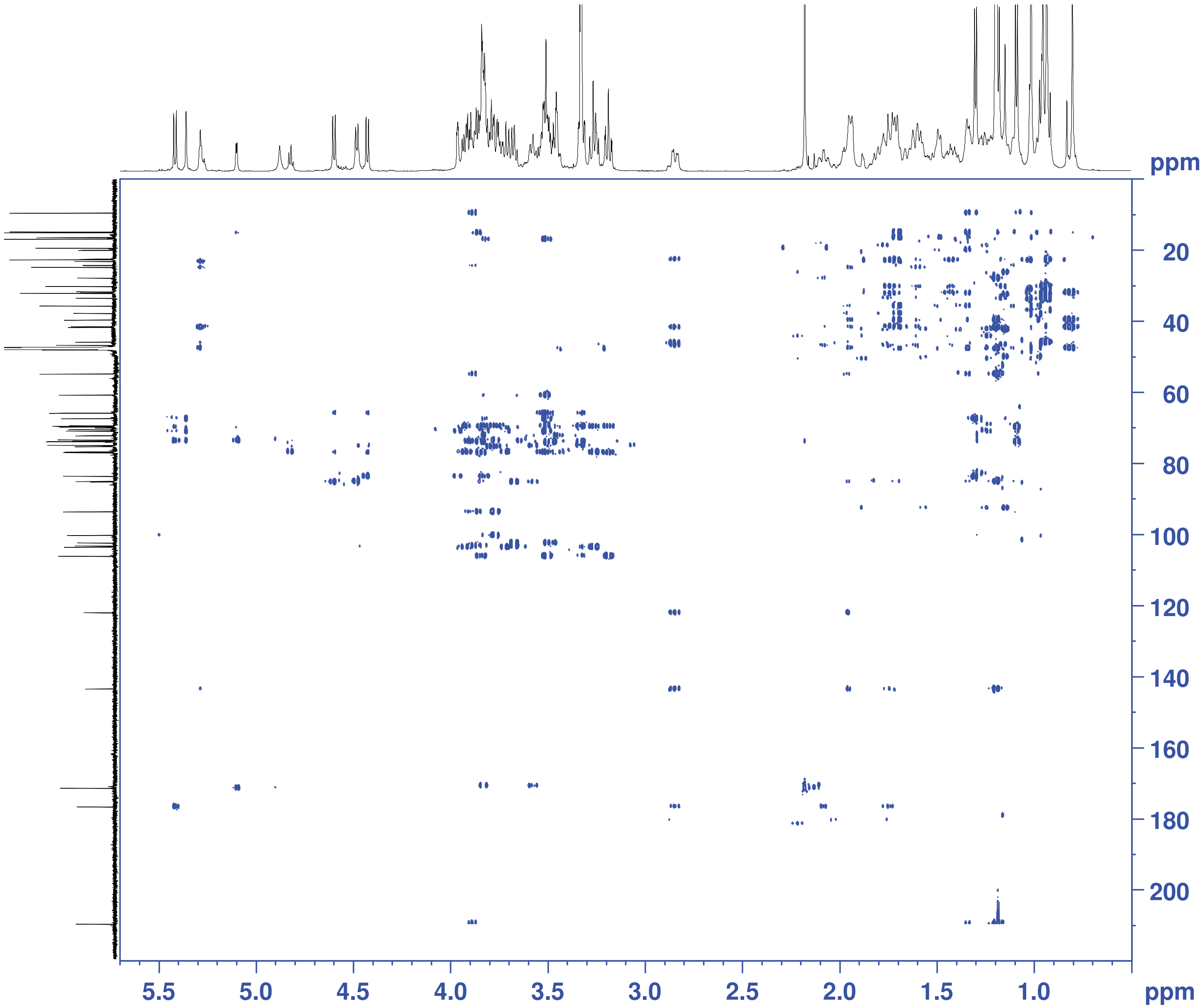

SO1406 HSQC (CD3OD, 600 MHz, 150 MHz)

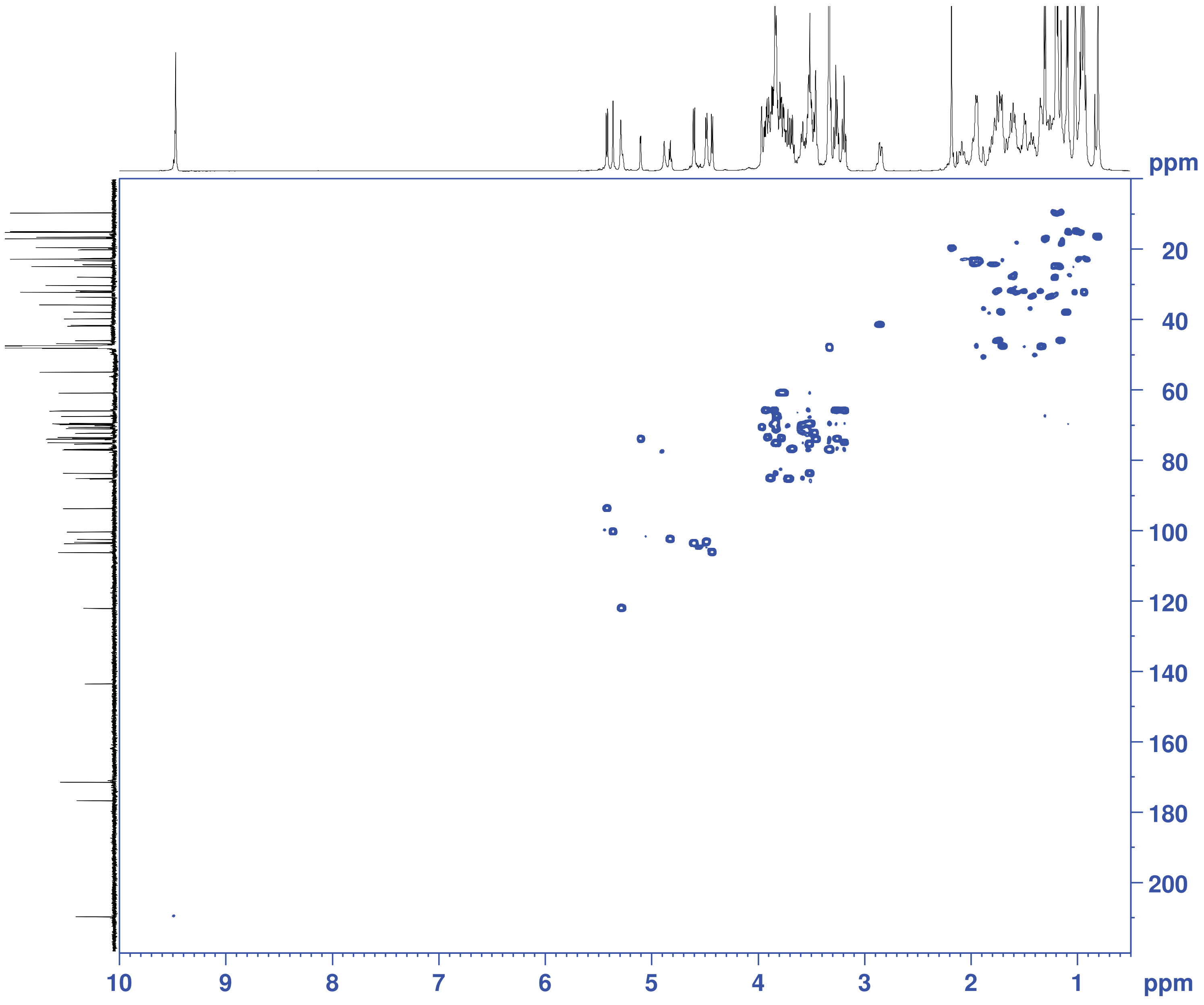

SO1406 HSQC (CD3OD, 600 MHz, 150 MHz)

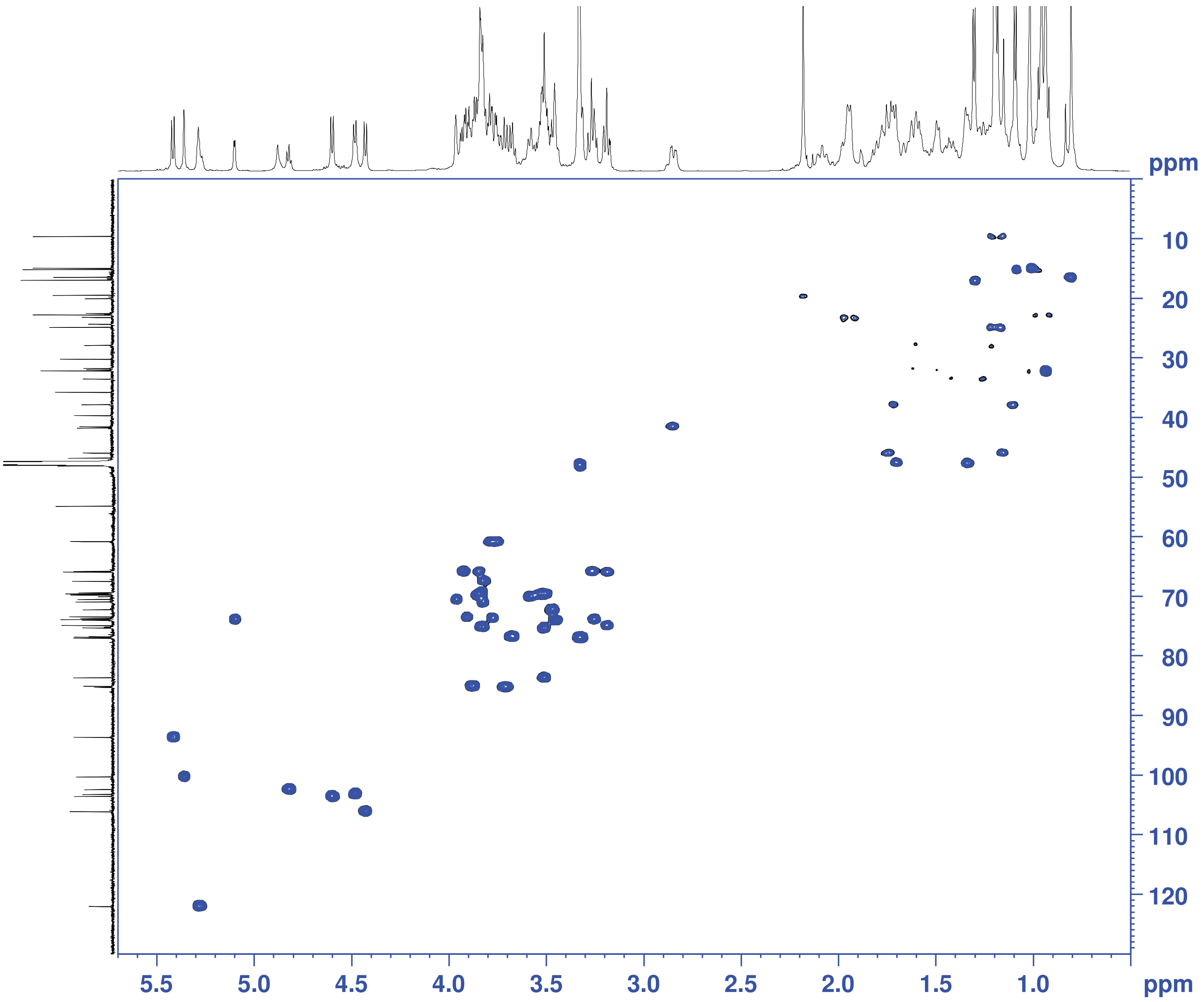

SO1726 COSY (C5D5N, 600 MHz)

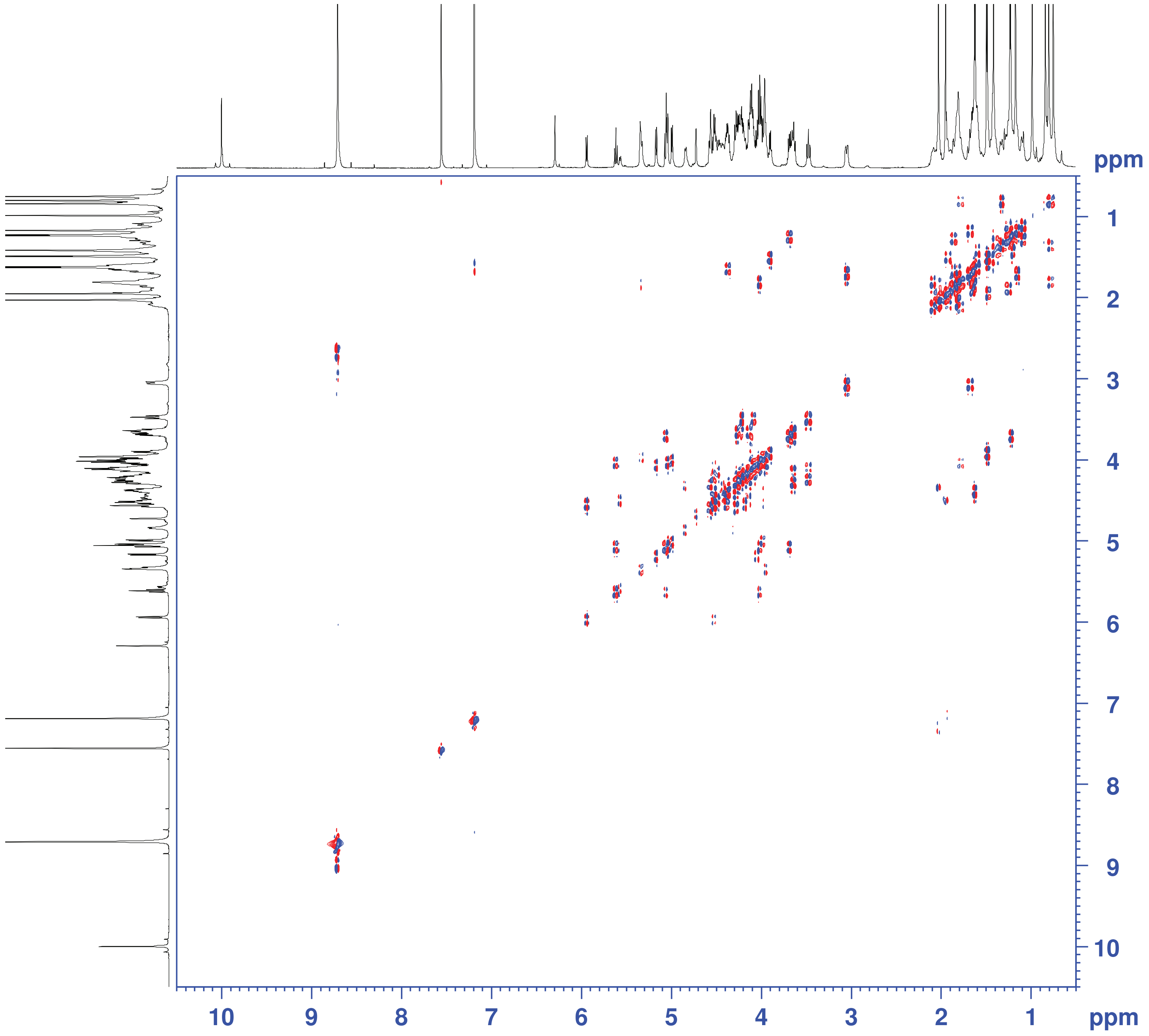

SO1726 COSY (C5D5N, 600 MHz)

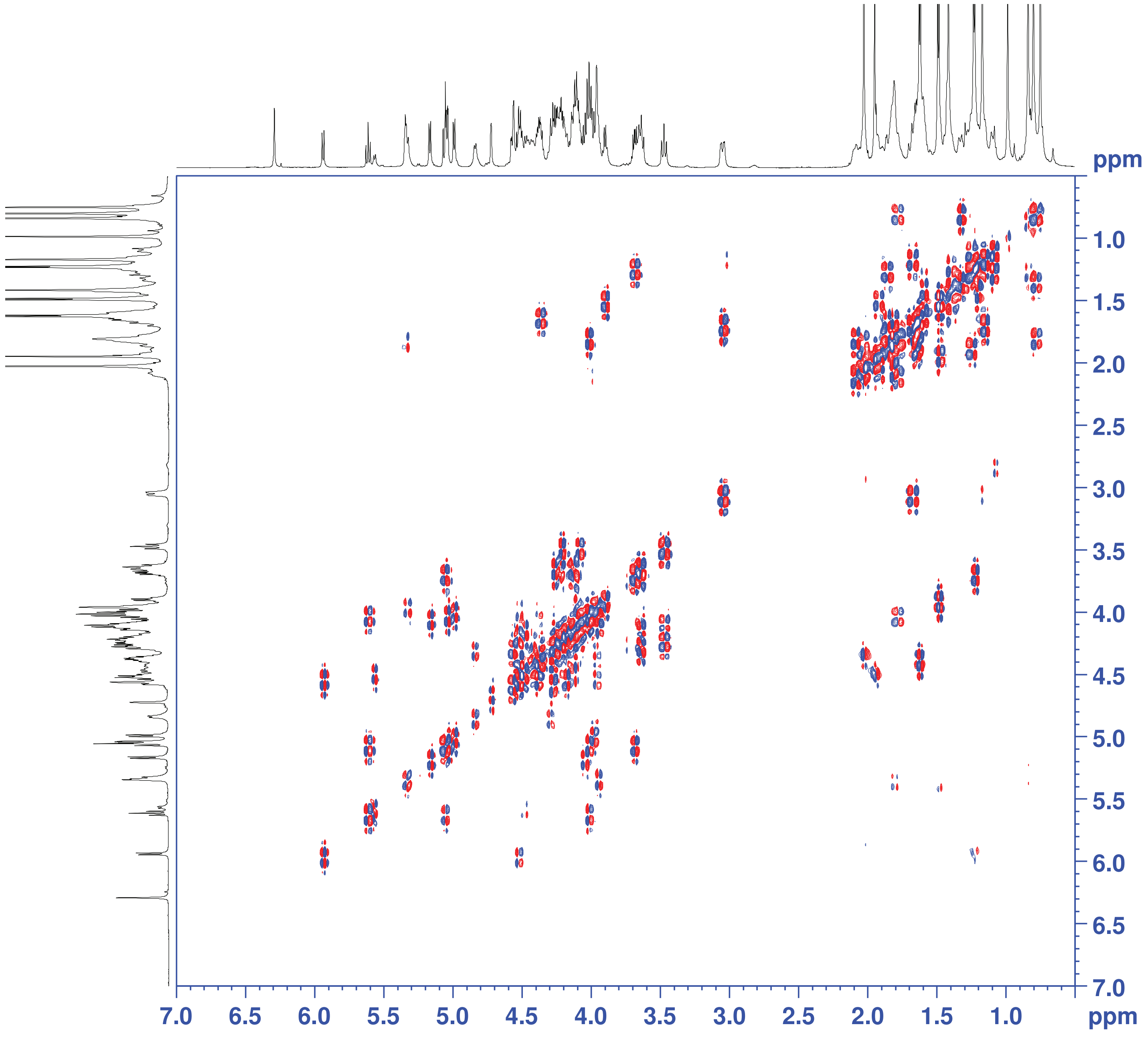

SO1726 TOCSY (C5D5N, 600 MHz)

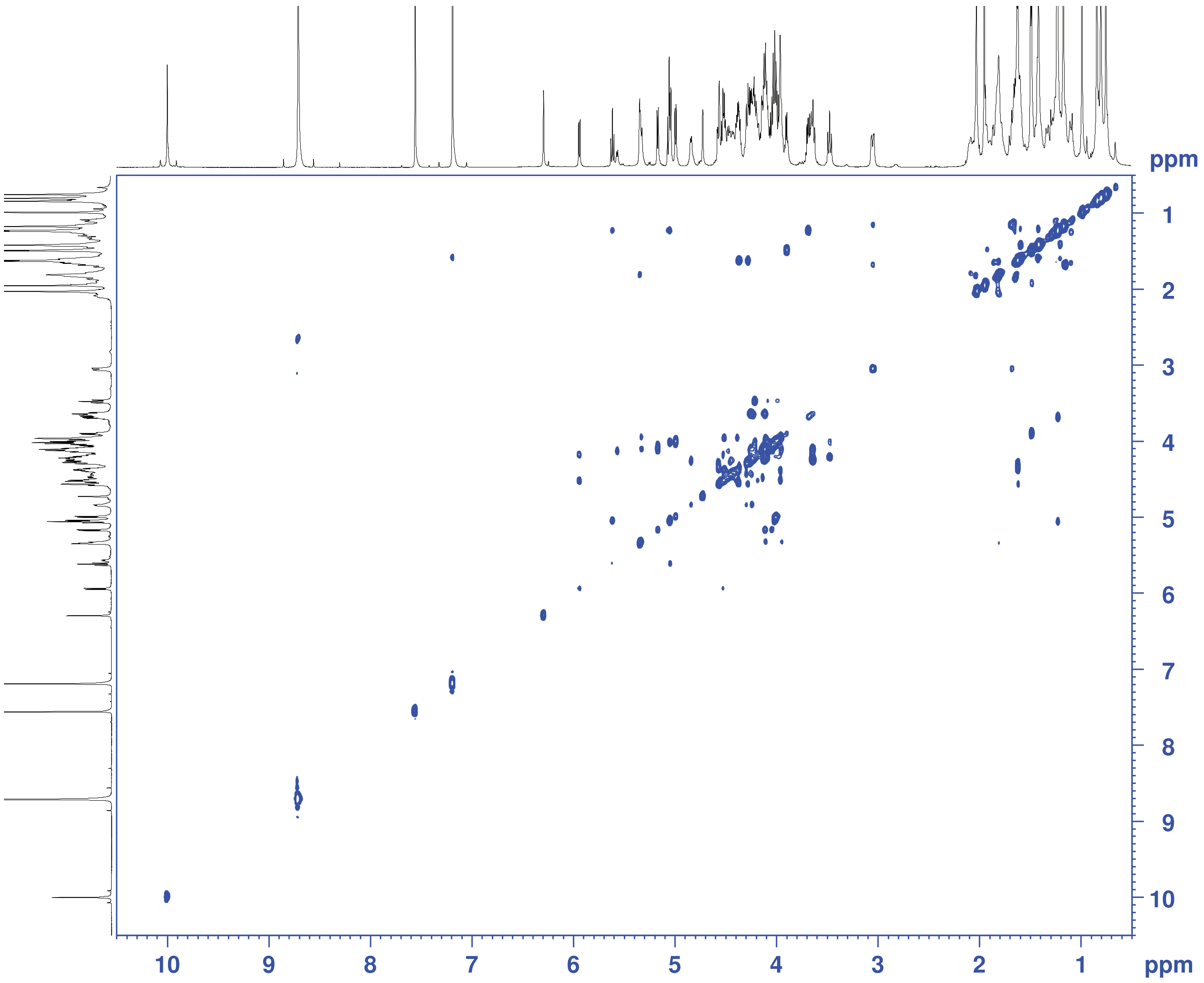

SO1726 TOCSY (C5D5N, 600 MHz)

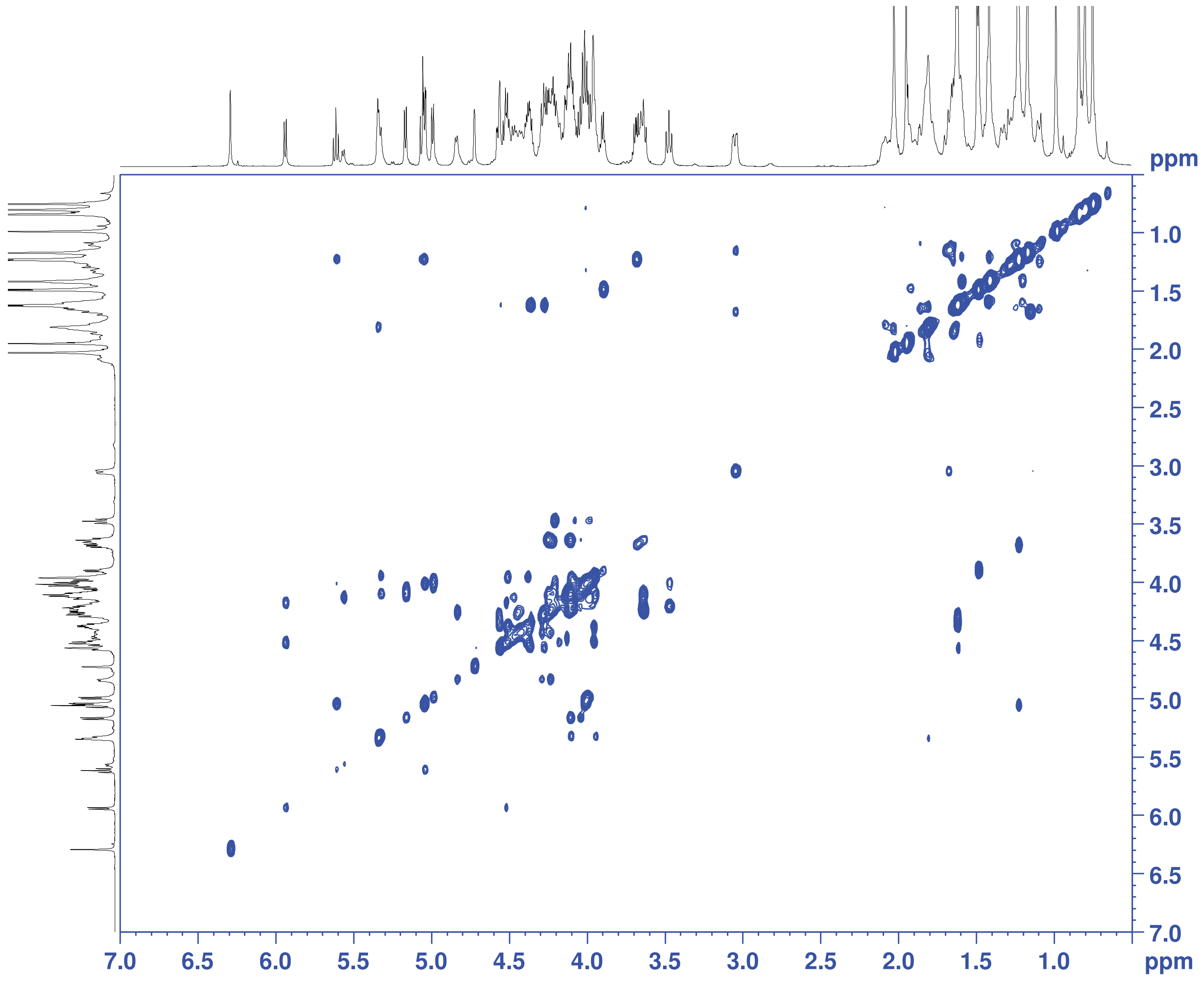

SO1726 HMBC (C5D5N, 600 MHz, 150 MHz)

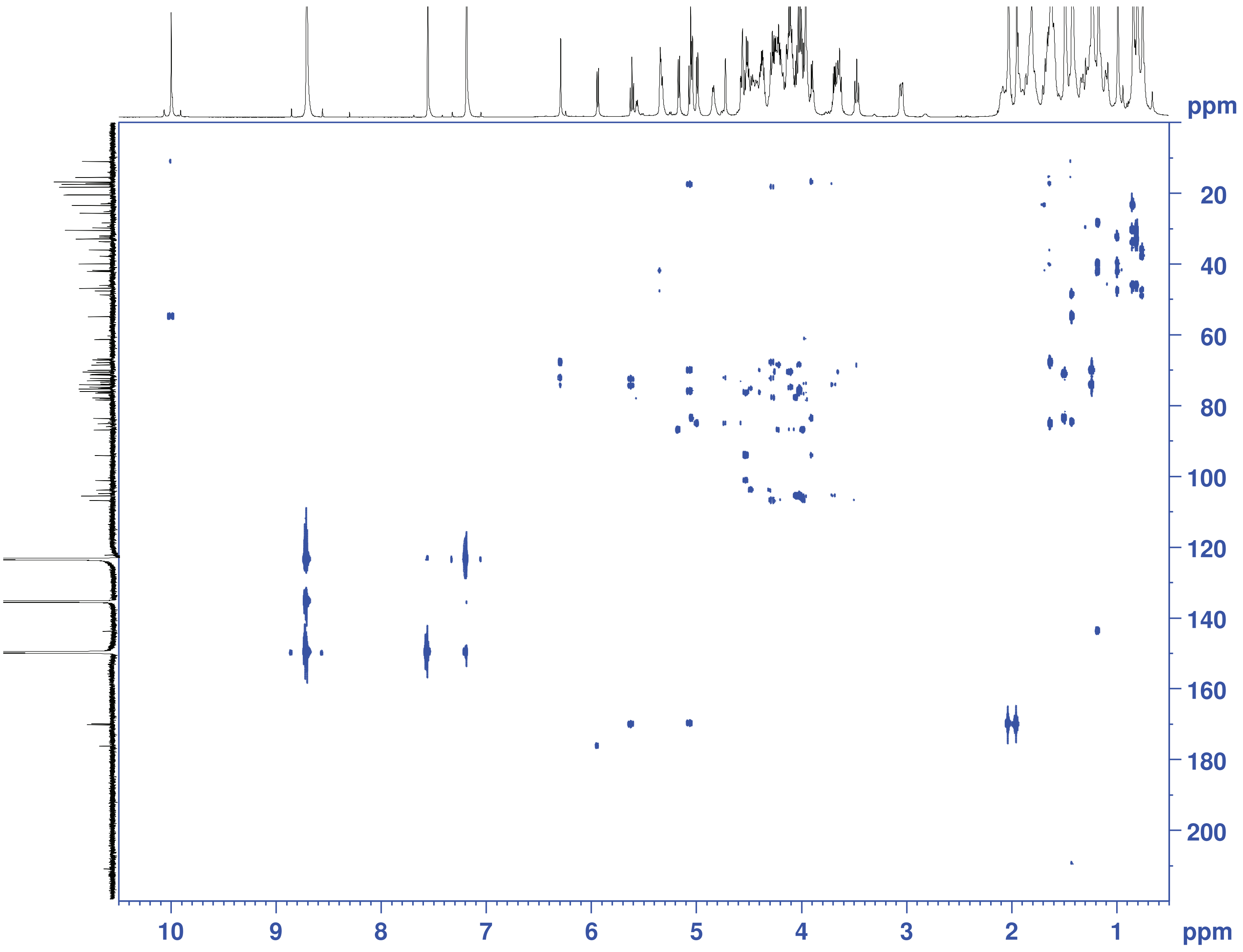

SO1726 HMBC (C5D5N, 600 MHz, 150 MHz)

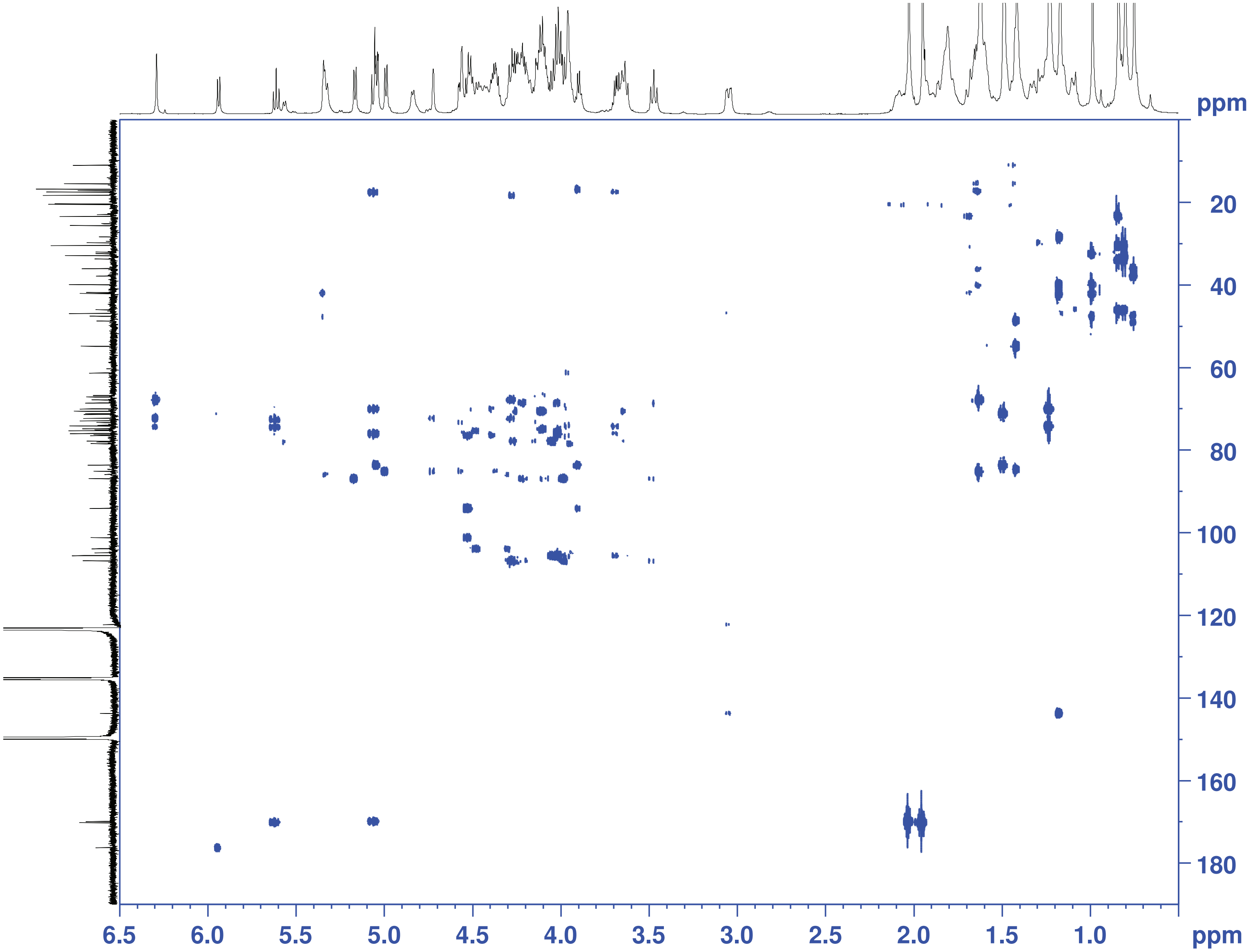

SO1726 HSQC (C5D5N, 600 MHz, 150 MHz)

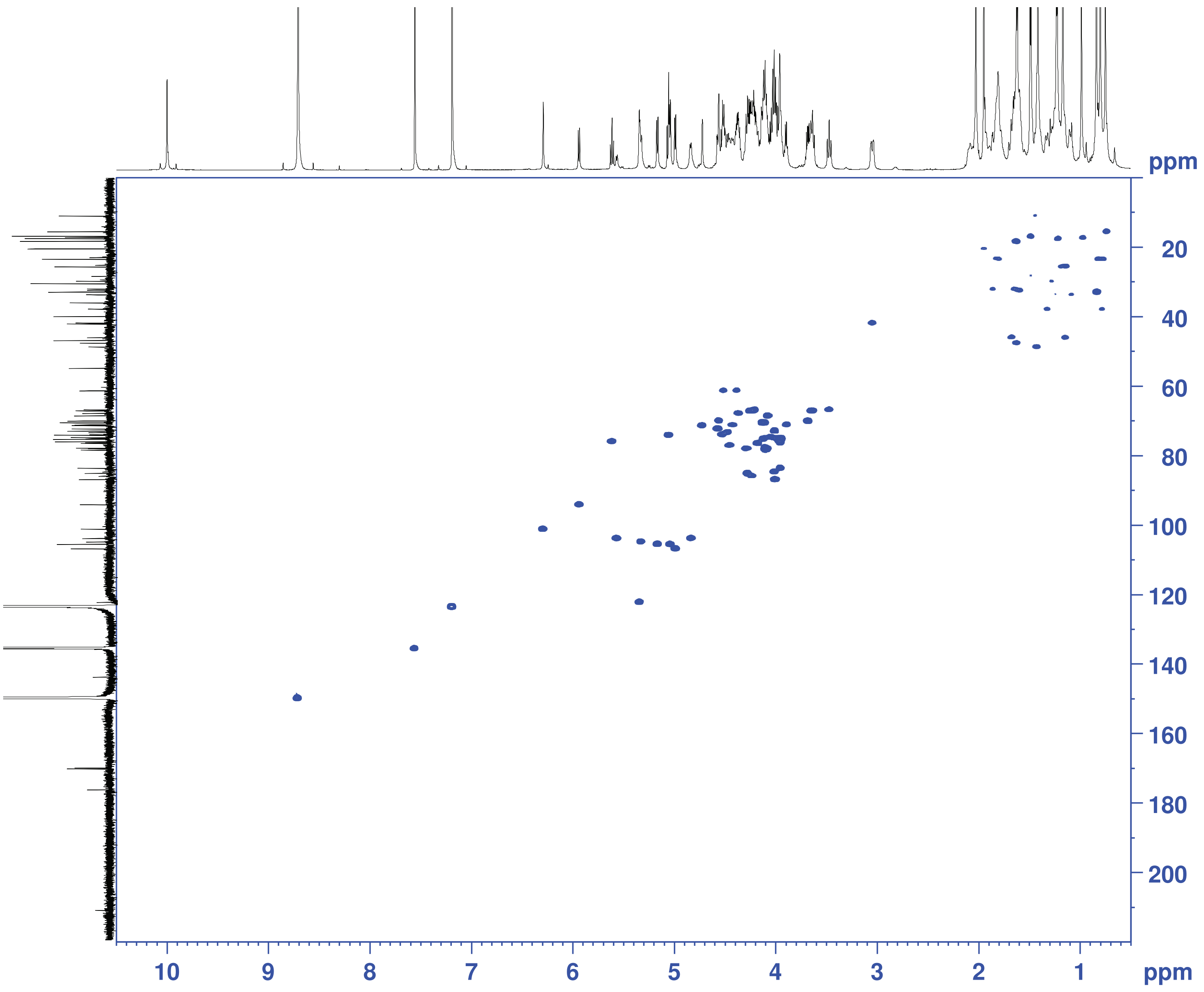

SO1726 HSQC (C5D5N, 600 MHz, 150 MHz)

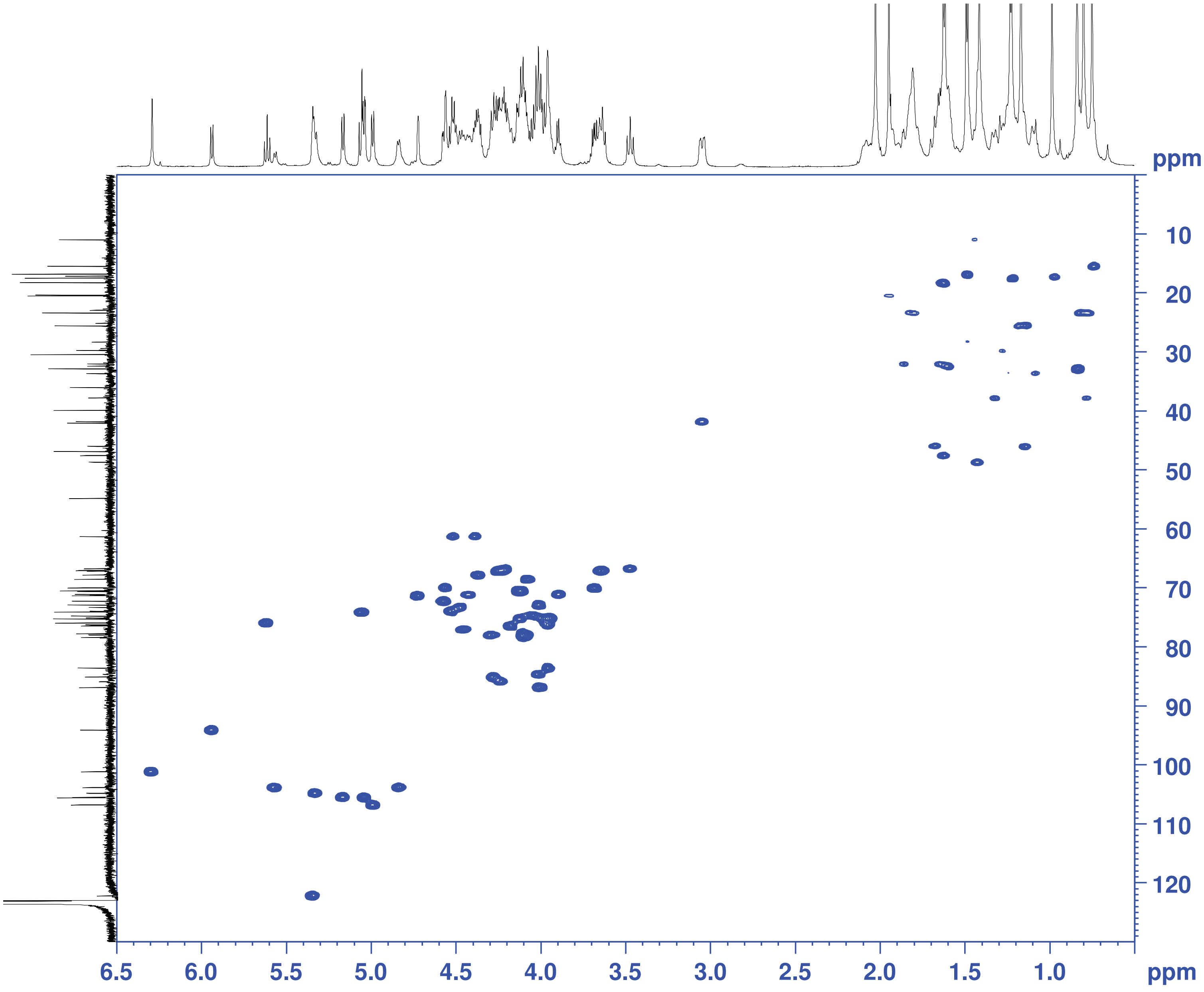

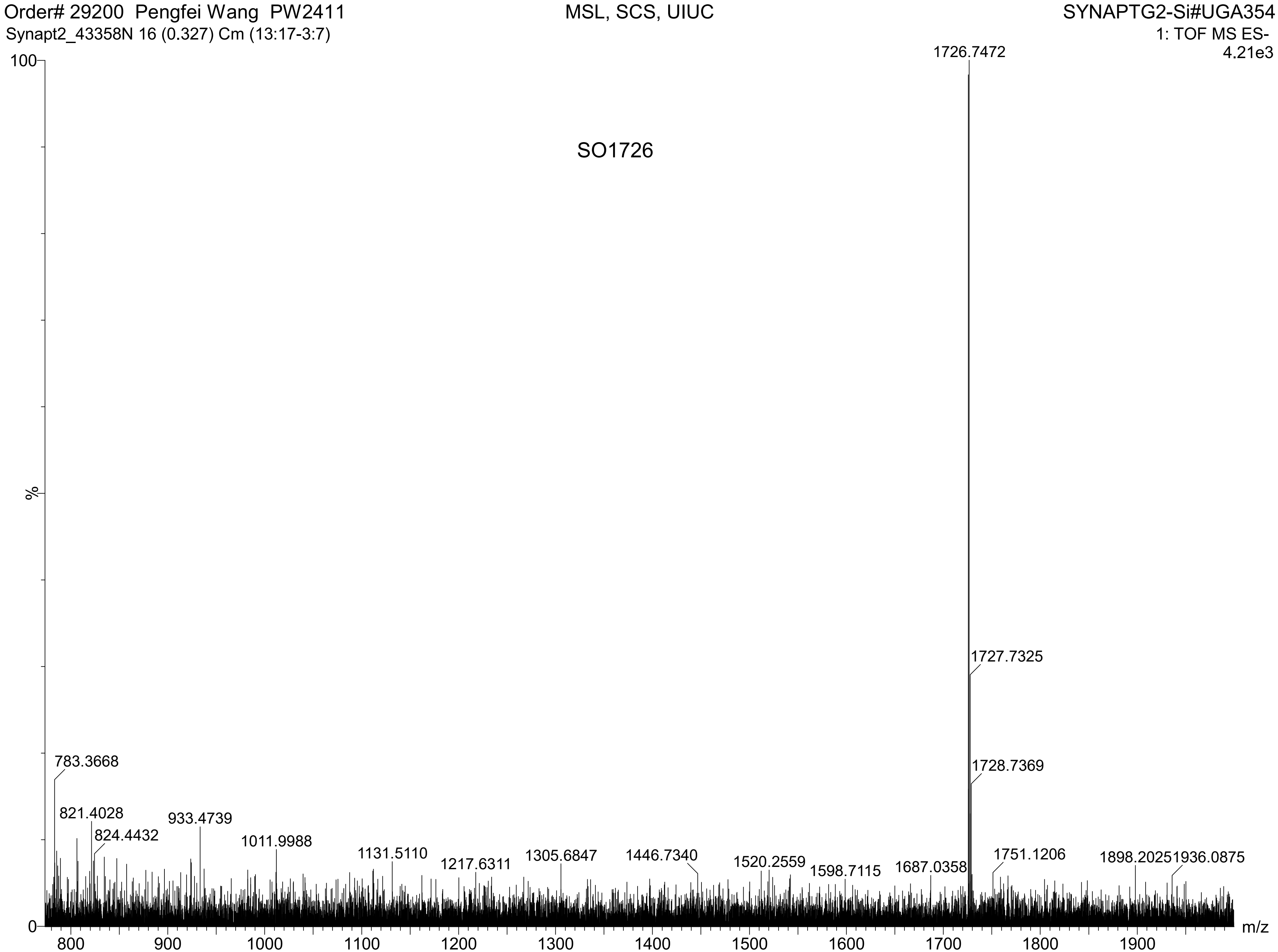

Order# 29200 Pengfei Wang PW2411
MSL, SCS, UIUC
SYNAPTG2-Si#UGA354
Synapt2_43358N 16 (0.327) Cm (13:17-3:7)
1: TOF MS ES-
4.21e3
1726.7472
SO1726
100
%
0
1727.7325
1728.7369
783.3668
821.4028
824.4432
933.4739
1011.9988
1131.5110
1217.6311
1305.6847
1446.7340
1520.2559
1598.7115
1687.0358
1751.1206
1898.2025
1936.0875
800
900
1000
1100
1200
1300
1400
1500
1600
1700
1800
1900
m/z

Order# 29203 Pengfei Wang PW2411 MSL, SCS, UIUC SYNAPTG2-Si#UGA354

Synapt2_43361N 14 (0.293) Cm (14:17-3:6) 1: TOF MS ES- 1.04e4

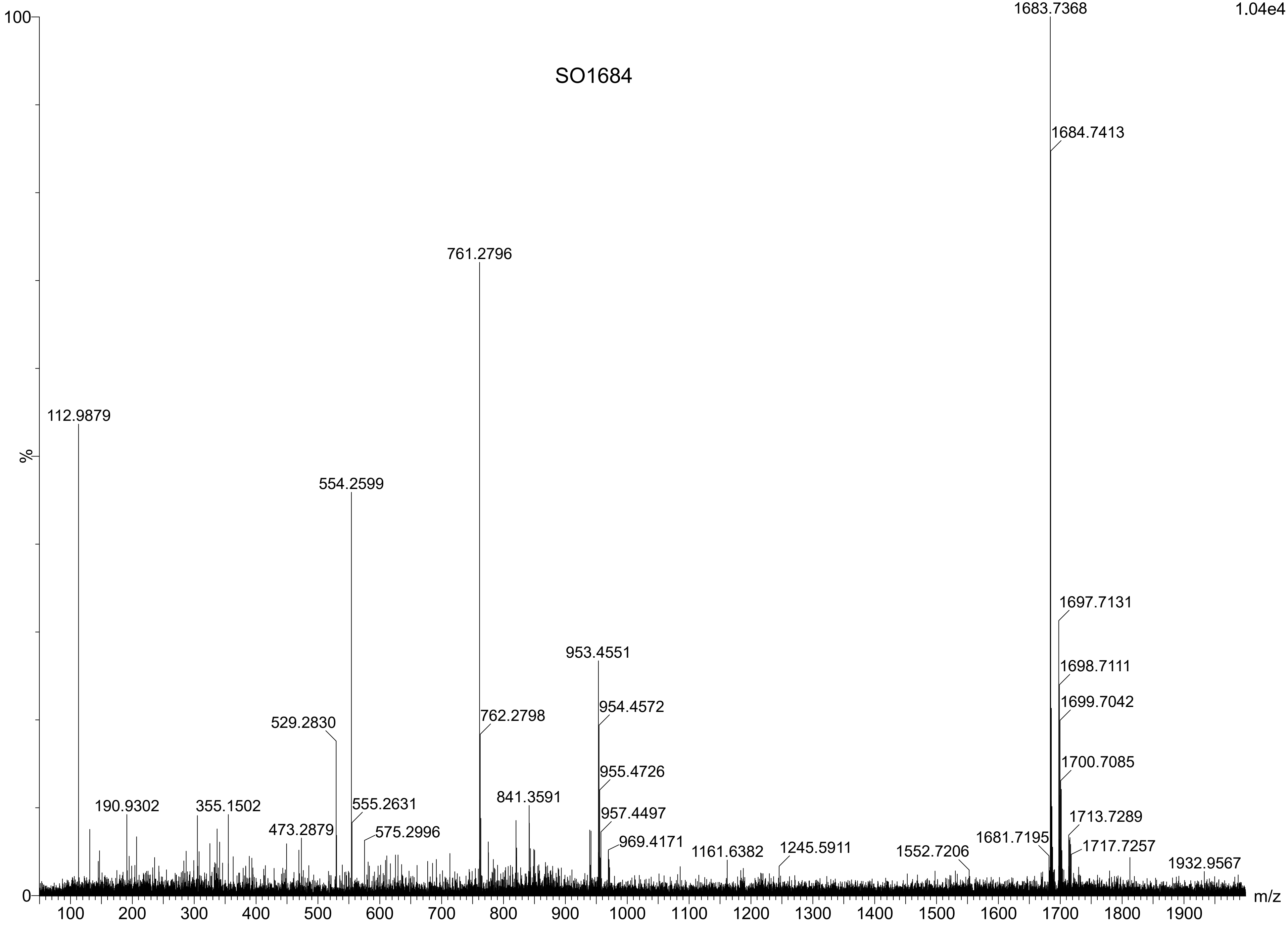

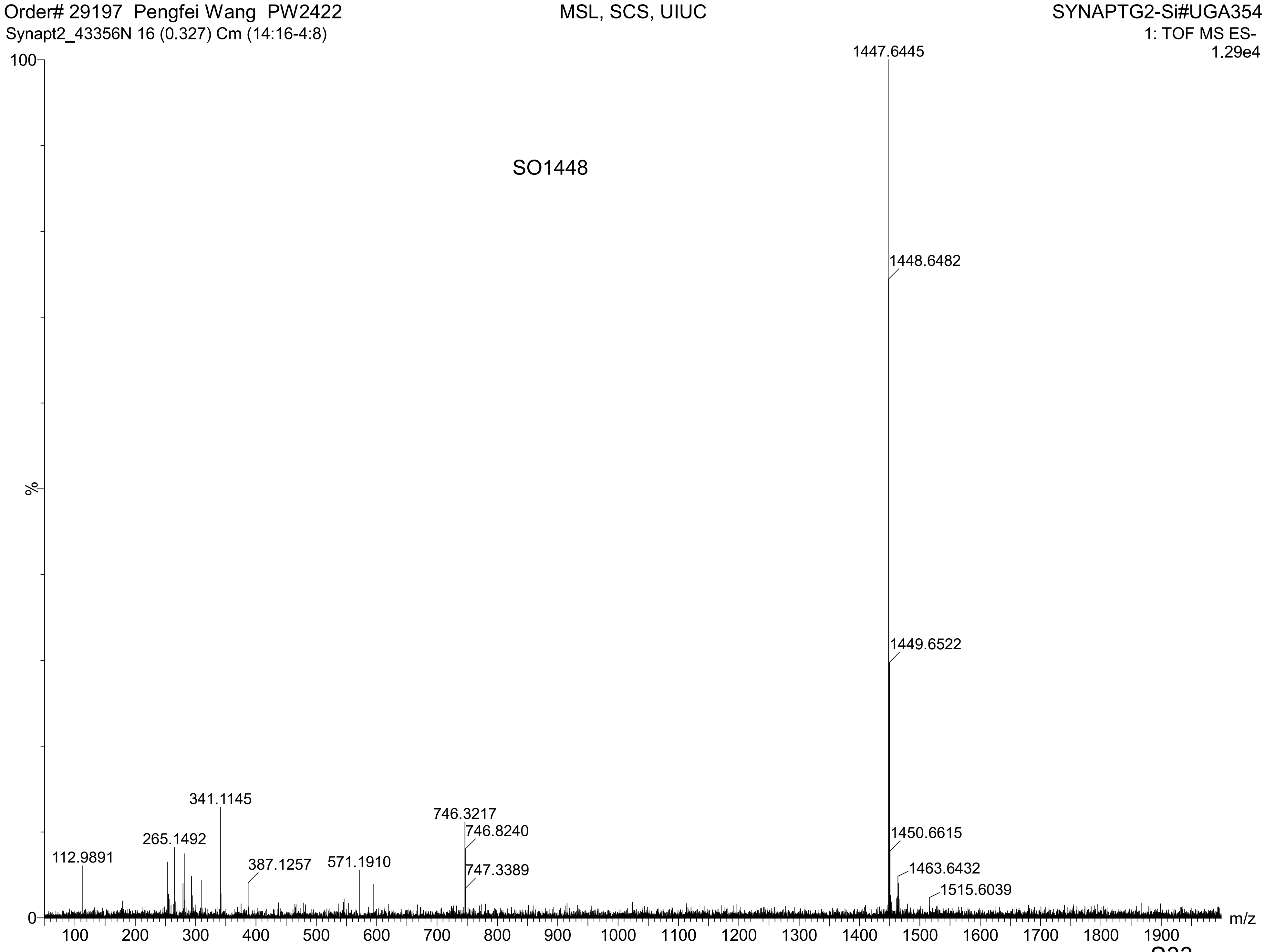

Order# 29197 Pengfei Wang PW2422
MSL, SCS, UIUC
SYNAPTG2-Si#UGA354
Synapt2_43356N 16 (0.327) Cm (14:16-4:8)
1: TOF MS ES-
1.29e4
SO1448
1447.6445
1448.6482
1449.6522
1450.6615
1463.6432
1515.6039
341.1145
265.1492
112.9891
387.1257
571.1910
746.3217
746.8240
747.3389
100
%
0
m/z
100
200
300
400
500
600
700
800
900
1000
1100
1200
1300
1400
1500
1600
1700
1800
1900

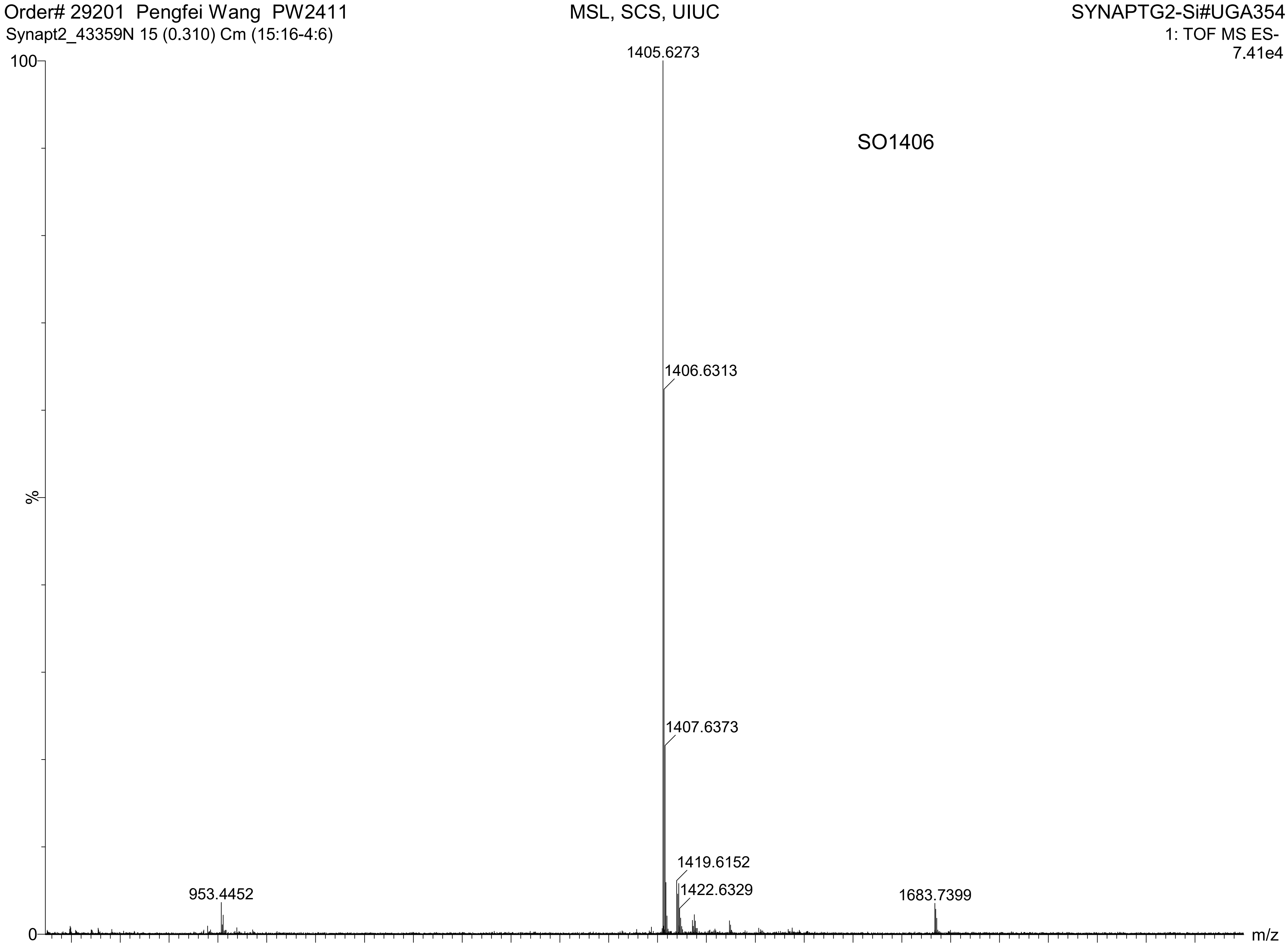

Order# 29201 Pengfei Wang PW2411
MSL, SCS, UIUC
SYNAPTG2-Si#UGA354
Synapt2_43359N 15 (0.310) Cm (15:16-4:6)
1: TOF MS ES-
7.41e4
SO1406
1405.6273
1406.6313
1407.6373
1419.6152
1422.6329
953.4452
1683.7399
100
%
0
800
900
1000
1100
1200
1300
1400
1500
1600
1700
1800
1900
m/z

SynergyFinder+ Report

1. Data tables

Drug Combiantion Meta Data

Block ID	Drug$_1$	Drug$_2$	Conc Unit$_1$	Conc Unit$_2$
1	Saporin	SO1406	ug/ml	ug/ml

Synergy Score Summary Table

Block ID	ZIP	Loewe	HSA	Bliss
1	27.42	33.48	34.98	26.8

2. Dose response curve

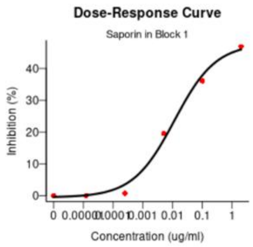

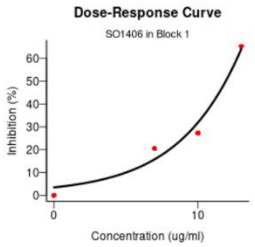

3. Dose response map

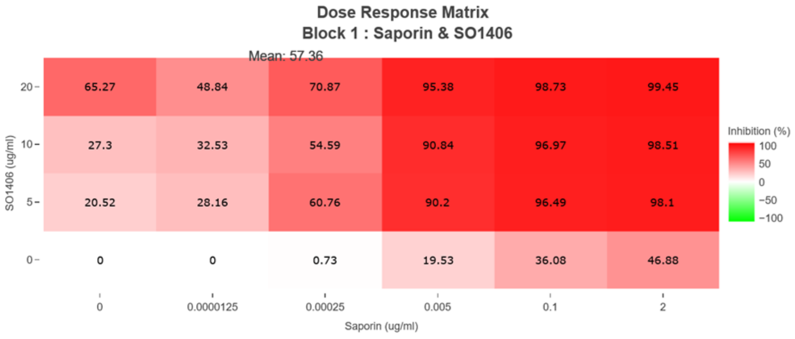

4. Synergy Scores

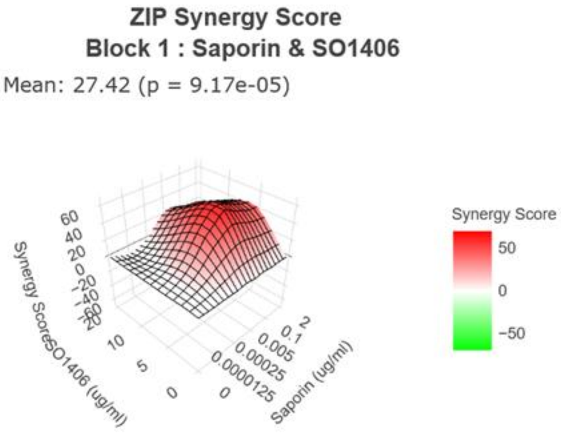

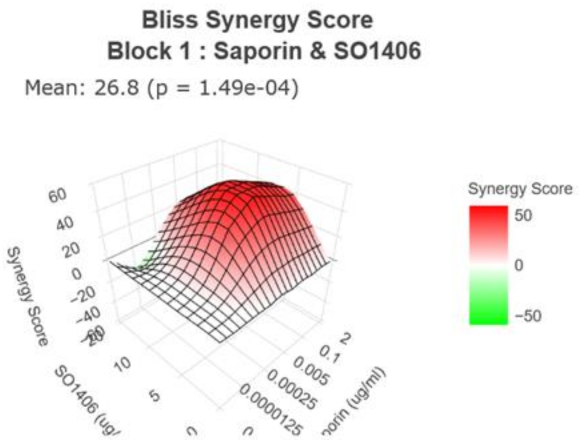

Supplement: Supplementary file 1 [file biomedicines-14-00626-s001.zip › biomedicines-4161171-supplementary.pdf]
